# Rapid Analysis of NAD and Other Phosphorylated Metabolites in Complex Biological Samples by Hydrophilic Interaction Liquid Chromatography Coupled with Tandem Mass Spectrometry

**DOI:** 10.1021/acs.analchem.6c00721

**Published:** 2026-04-08

**Authors:** Adela Pravdova, Maximilian Kleinert, John Henderson, Eleni Kafkia, David Pladevall-Morera, Caio Y. Yonamine, Jonas T. Treebak, Tetiana Brodiazhenko, Ilya Terenin, Jan Jakub Zylicz, Thomas Moritz, Ondrej Hodek

**Affiliations:** 1 Department of Forest Genetics and Plant Physiology, Swedish University of Agricultural Sciences, Umeå 907 36, Sweden; 2 Institute of Organic Chemistry and Biochemistry of CAS, 160 00 Prague, Czech Republic; 3 Department of Analytical Chemistry, Faculty of Science, Charles University, 110 00 Prague, Czech Republic; 4 Swedish Metabolomics Centre, Umeå 907 36, Sweden; 5 Department of Molecular Physiology of Exercise and Nutrition, 14927 German Institute of Human Nutrition (DIfE), Potsdam-Rehbruecke, Nuthetal 14558, Germany; 6 German Center for Diabetes Research (DZD), Munich 85764, Germany; 7 Novo Nordisk Foundation Center for Basic Metabolic Research, Faculty of Health and Medical Sciences, 554063 University of Copenhagen, Blegdamsvej 3B, Copenhagen N 2200, Denmark; 8 Novo Nordisk Foundation Center for Stem Cell Medicine, reNEW, University of Copenhagen, Copenhagen 2200, Denmark; 9 684400 Icosagen Cell Factory OÜ, Õssu 61713, Estonia; 10 SciLifeLab, 5193 Lund University, Lund 221 00, Sweden

## Abstract

Nucleotides and coenzymes play critical roles in energy metabolism and cellular signaling and as building blocks of nucleic acids. This work addresses the challenges in the measurement of the phosphorylated metabolites using hydrophilic interaction liquid chromatography coupled with mass spectrometry, which facilitates the separation and detection of polar metabolites. Here, we present optimized HILIC-MS/MS methods for rapid analysis of polar metabolites including nucleotides and their derivatives in complex biological matrices, such as murine adipose, skeletal, and liver tissues, human plasma, and bacteria. The developed methodologies enable separation of key nucleotides and other phosphorylated metabolites within 6 min and cofactors such as NAD^+^, NADH, NADP^+^, and NADPH within 4 min. Validation of these methods demonstrated high accuracy, precision, and sensitivity and stresses the substantial impact of matrix effects. The applicability of the methods was also tested on ^13^C-labeling experiments with mouse pluripotent stem cells. Additionally, sample pretreatment techniques, such as liquid–liquid extraction and solid-phase extraction, were evaluated as a tool to decrease the negative impact of matrix effects in complex samples. This work enhances the analytical capabilities for nucleotide quantification in metabolomics, facilitating the study of metabolic pathways and disease markers.

## Graphical abstract

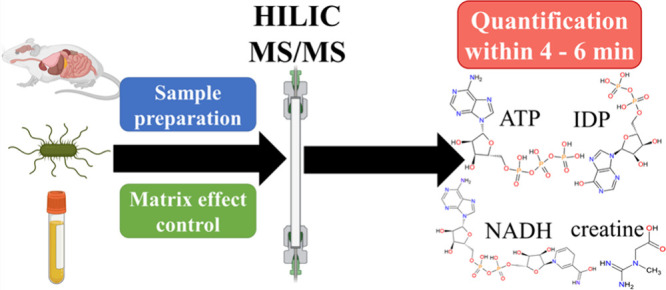

## Introduction

Metabolomics is a relatively new and rapidly developing field focused on identifying and quantifying metabolome components in biological systems.^1–3^ Currently, more than 200,000 endogenous metabolites are known.^1^ A notable subset of these are nucleotides—small, polar molecules involved in numerous biochemical processes in prokaryotes, plants, animals, and fungi. Phosphorylated metabolites such as nucleotides and coenzymes play essential roles in cellular metabolism.^4–6^ Adenosine triphosphate (ATP), cytidine triphosphate (CTP), guanosine triphosphate (GTP), and uridine triphosphate (UTP) serve as energy sources.^4^ Nucleotide derivatives like cyclic AMP (cAMP), cyclic diadenosine monophosphate (c-di-AMP), and cyclic diguanosine monophosphate (c-di-GMP), along with alarmones ppGpp and pppGpp, act as second messengers with key regulatory functions.^6,7^ Nicotinamide adenine dinucleotide (NAD^+^) and its reduced and phosphorylated forms (NADH, NADP^+^, and NADPH) are vital metabolites present in all living cells. They act as electron carriers and redox cofactors in central carbon metabolism, biosynthesis, antioxidant defense, and also play roles in cell signaling and transduction.^8,9^

Therefore, the accurate and reproducible measurement of phosphorylated metabolites is critical in metabolomics. Liquid-phase separation methods, such as capillary electrophoresis (CE)^10,11^ and liquid chromatography (LC), are commonly used to analyze nucleotides and other polar metabolites. CE enables direct detection of NAD metabolites^12^ or detection following online enzymatic assays with UV–vis detection.^13^ UV–vis has also been used with LC to analyze NAD metabolites.^14,15^ However, it offers lower specificity and insufficient sensitivity compared with tandem mass spectrometry (MS/MS). MS detection with LC requires low buffer concentrations in the mobile phase, which makes efficient separation of phosphorylated metabolites challenging (e.g., NAD^+^, NADH, NADP^+^, NADPH). This has led to three main LC-MS strategies for polar metabolite analysis: ion-pair reversed-phase chromatography,^16,17^ ion exchange liquid chromatography,^18,19^ and hydrophilic interaction liquid chromatography (HILIC).^20,21^ HILIC avoids ion-pairing reagents, and when medronic acid is added to the mobile phase as a passivation agent, it prevents tailing of metal-sensitive compounds such as phosphorylated metabolites.^22^ The advantage of HILIC separation over other techniques is the ability to modulate retention by adjusting pH and buffer salt concentration in the mobile phase.^21,23^ HILIC enables separation of both charged and neutral molecules, and when combined with electrospray MS detection, it is widely used for nucleotide quantification in complex systems.^10,21,22,24^ Recent studies have applied HILIC-MS/MS for quantifying nucleotides in plasma,^25^ infant formulas,^26^ and yeast^27^ or for detecting nucleotides in bacterial cells, including alarmones ppGpp and pppGpp.^6^ Even though HILIC-MS setups are also suitable for high-throughput workflows,^28^ existing HILIC methods for analyzing NAD and other nucleotides either lack high-throughput capacity due to long analysis times (15–30 min)^29,30^ or suffer from insufficient selectivity in high-throughput methods.^28^

We developed fast HILIC methods for analyzing nucleotides and their derivatives using liquid chromatography with tandem mass spectrometry in complex biological matrices including murine liver, skeletal muscle, adipose tissue, human plasma, and bacteria. A specific HILIC method achieved baseline separation of NAD^+^, NADH, NADP^+^, and NADPH within 4 min and allowed absolute quantification in murine liver, skeletal muscle, and white adipose tissue extracts. The methods were validated for accuracy, precision, sensitivity, linearity, carry-over, matrix effects, recovery, and stability. During optimization, multiple HILIC stationary phases were tested with an optimized gradient.

Highly complex biological samples often require pretreatment to reduce matrix effects and/or preconcentrate analytes.^2,24^ These steps remove salts, phospholipids, or proteins that affect metabolite retention or suppress the MS response. Proteins can irreversibly adsorb to the stationary phase, reducing column efficiency or causing clogging. Salts and phospholipids significantly contribute to matrix effects, suppressing or enhancing ionization.^10,31^ Proper pretreatment is crucial for reliable, accurate results, as metabolic disease markers are often present at low concentrations and may be suppressed by the sample matrix.^10^ Typically, nucleotides are separated from proteins by precipitation. Additional techniques like liquid–liquid extraction (LLE)^10,11^ or solid-phase extraction (SPE)^10,31^ further reduce matrix complexity, enabling selective nucleotide isolation and concentration. Although LLE and SPE are time-consuming, they efficiently eliminate interfering substances and minimize matrix effects. Therefore, LLE and SPE were evaluated in this study as pretreatment methods for human plasma to eliminate the main contributors to matrix effects.

## Material and Methods

### Chemicals and Reagents

All chemicals and standards are listed in Supporting Information Table S1.

### Preparation of Standard Solutions

All preparation procedures for phosphorylated metabolites and lipid solutions are described in the Supporting Information.

### Stability Testing of Standard Nucleotides

The detailed procedure is described in the Supporting Information.

### Sample Preparation of Human Plasma, Murine Liver, Adipose, Skeletal Muscle Tissue, Bacterial Extracts, and Liver for Quantification of NAD Metabolites

All detailed preparation procedures are described in the Supporting Information.

### Sample Preparation of Mouse Pluripotent Stem Cells Treated with ^13^C_6_-Glc for Labeling Experiments

All detailed preparation procedures are described in the Supporting Information.

### LC-MS Method for Separation of NAD Metabolites

Separation and quantification of NAD metabolites were performed on an Agilent 1290 UHPLC system coupled with an Agilent 6495D triple quadrupole mass spectrometer (Agilent, USA). A 3 μL sample extract (10× diluted with 80% aqueous acetonitrile) was injected onto an iHILIC-Fusion column (SS, 30 × 2.1 mm, 1.8 μm; HILICON AB, Sweden). The mobile phases were as follows: (A) 10 mM ammonium acetate in water with 5 μM medronic acid and (B) 10 mM ammonium acetate in 90% aqueous acetonitrile. The column was operated at 0.6 mL/min using the following gradient: 0 min (85% B), 2.5 min (70% B), 2.7 min (30% B), 3 min (30% B), 3.01 min (85% B), and 4 min (85% B). Column and autosampler temperatures were maintained at 40 and 4 °C, respectively. Electrospray ionization in positive mode was used with the following source parameters: +4.0 kV ion spray voltage, 150 °C gas temperature, 11 L/min drying gas, 30 psi nebulizer pressure, 400 °C sheath gas temperature, 12 L/min sheath gas flow, and 380 V fragmentor voltage. The system operated in multiple reaction monitoring (MRM) mode, and MRM transitions for NAD metabolites were optimized using an Agilent MassHunter Optimizer.

### LC-MS Method for Separation of Nucleotides

Targeted analysis of nucleotides and their derivatives was performed using an Agilent 1290 UHPLC system coupled with an Agilent 6495D triple quadrupole mass spectrometer. UV detection was employed during stability studies, monitoring nucleotide triphosphates at 248, 254, 260, 262, and 271 nm. The following HILIC columns were tested: iHILIC-(P) Classic (30 × 2.1 mm, 5 μm), iHILIC-(P) Classic PEEK (50 × 2.1 mm, 5 μm), iHILIC-Fusion SS (50 × 2.1 mm, 1.8 μm), iHILIC-Fusion­(+) SS (50 × 2.1 mm, 1.8 μm), iHILIC-Fusion­(P) PEEK (50 × 2.1 mm, 5 μm) with guard column (all from HILICON AB, Sweden), and ACQUITY BEH Z-HILIC (50 × 2.1 mm, 1.7 μm), BEH Amide (50 × 2.1 mm and 30 × 2.1 mm, 1.7 μm) from Waters (USA). The column temperature was 40 °C. Mobile phase A consisted of 10 mM ammonium acetate with 5 μM medronic acid in water (pH 6.8), while phase B contained 10 mM ammonium acetate in 90% aqueous acetonitrile. The flow rate was set at 0.35 mL/min for 5 cm columns and 0.6 mL/min for 3 cm columns, with an injection volume of 3 μL. The gradient for the 5 cm column was: 0 min (85% B), 5 min (60% B), 7 min (30% B), 8 min (30% B), 9 min (85% B), and 15 min (85% B); and for the 3 cm column: 0 min (95% B), 0.5 min (85% B), 3 min (70% B), 3.9 min (70% B), 4.0 min (50% B), 4.79 min (50% B), 4.8 min (95% B), and 6 min (95% B). Electrospray ionization and dynamic MRM (dMRM) mode were used, with the following source parameters: +4 kV (positive mode), −3.5 kV (negative mode), gas temperature of 200 °C, drying gas flow of 11 L/min, nebulizer pressure of 30 psi, sheath gas temperature of 375 °C, and sheath gas flow of 12 L/min. MRM transitions were optimized using Agilent MassHunter Optimizer, and data were processed using MassHunter Qualitative Analysis 10.0 and Quantitative Analysis B.07.00.

### LC-MS Method for Lipid Analysis

All parameters used for lipidomic analysis are provided in the Supporting Information.

### Method Validation for Separation of NAD Metabolites, Nucleotides, and Their Derivates

The method for quantification of NAD metabolites was evaluated in terms of sensitivity, linearity, accuracy, precision, carry-over, repeatability of peak areas, matrix effects, recovery, and stability. The validation process and the calculations are described in detail in the Supporting Information.

### Liquid–Liquid Extraction Sample Pretreatment

A mixture of lipid and nucleotide standards or human plasma extract was added to a 1.5 mL Eppendorf tube containing 500 μL of water and vortexed for 10 s. Subsequently, 500 μL of ethyl acetate were added to form a two-phase system followed by vortexing for 30 s and centrifugation at 500*g* for 30 s to separate the phases. The upper phase was transferred to a clean tube, and both phases were evaporated by using a vacuum concentrator. Prior to LC-MS analysis, samples were reconstituted in 50 μL of 50% aqueous methanol for nucleotide analysis or in 40 μL of isopropyl alcohol/methanol (1:1, v/v) for lipidomic analysis.

### Solid-Phase Extraction Sample Pretreatment

ISOLUTE C18 SPE cartridges (100 mg, Biotage Sweden AB) were conditioned with 200 μL of acetonitrile and equilibrated twice with 200 μL of 0.1% TFA in 5% acetonitrile. A standard mixture or plasma extract 10× diluted with water was applied, and the flow-through was collected. The cartridge was then washed twice with 500 μL of 0.1% TFA in 5% acetonitrile and finally with 500 μL of methanol/isopropyl alcohol (1:1, v/v), collecting each fraction. Samples were evaporated and reconstituted in 50 μL of 50% aqueous methanol (nucleotide analysis) or 40 μL of isopropyl alcohol/methanol (1:1, v/v) for lipidomic analysis followed by LC-MS. For HILIC-SPE, iSPE-HILIC cartridges (50 mg, HILICON AB) were conditioned with 500 μL of water and 500 μL of acetonitrile. Standards or plasma extracts were 5× diluted with 90% methanol and loaded and allowed to interact for 1 min, and the flow-through was collected. The cartridge was washed with 500 μL of acetonitrile, and then the nucleotides were eluted twice with 500 μL of water. Each fraction was collected, evaporated, and reconstituted as described above for subsequent LC-MS analysis.

## Results and Discussion

The study’s initial phase focused on optimizing the mobile phase composition, particularly pH, to evaluate its effect on HILIC chromatographic performance. Ammonium acetate and ammonium formate were compared at neutral pH and pH 9.0, both at 10 mM and 5 μM medronic acid. Since ammonium formate did not improve separation or peak shape, only ammonium acetate was used further. Mobile phase composition was then tested with varying pH (2.6, 4.0, 6.8, and 9.4), keeping ammonium acetate and medronic acid concentrations constant at 10 mM and 5 μM, respectively. The optimization was conducted on 5 stationary phases – iHILIC - (P) Classic, iHILIC-Fusion, iHILIC-Fusion (+), iHILIC-Fusion­(P), and BEH Z-HILIC. The performance of the tested columns was compared based on the quality score accounting for signal-to-noise ratio (*S*/*N*) and peak tailing factor of each tested compound on the stationary phase according to the equation^32^ in Table S2, where the scoring system is explained. The column comparison based on the quality score shows that the acidic pH (2.8 and 4.0) deteriorates the retention and sensitivity of nucleotide triphosphates on all columns (Figure S1). The iHILIC-(P) Classic stationary phase offered the best overall scoring among the tested columns and there were no substantial differences between the alkaline and neutral pH. To extend the column lifetime, a neutral pH of the mobile phase was used throughout the method development. The NAD^+^ cofactors were analyzed on the iHILIC-Fusion stationary phase as it offers baseline separation of these metabolites.

All metabolites were extracted by using 80% aqueous methanol with 0.1 M formic acid followed by neutralization with ammonium bicarbonate.

### Optimization of the LC-MS Method for Separation of NAD Metabolites

Collision energies for all MRM transitions of NAD metabolites were optimized through the flow injection analysis of 5 μM standards dissolved in 50% aqueous methanol (Table S3). Four MRM transitions were selected and tested in the on-column experiments. The transition with the highest signal-to-noise ratio was further used for analysis of real samples. The initial column screening for separation of the NAD metabolites was conducted on two stationary phases (i) iHILIC-(P) Classic, PEEK, 50 × 2.1, 5 μm and (ii) iHILIC-Fusion, SS, 50 × 2.1, 1.8 μm. Compared with iHILIC-(P) Classic, iHILIC-Fusion provided a better selectivity with baseline separation of the four metabolites (Figure S2). To accelerate the total analysis time, the separation was optimized on a shorter column – iHILIC-Fusion, SS, 30 × 2.1, 1.8 μm, which helped to speed up the analysis from 15 to 4 min while retaining sufficient chromatographic resolution (Figure 1).

**Figure 1:**
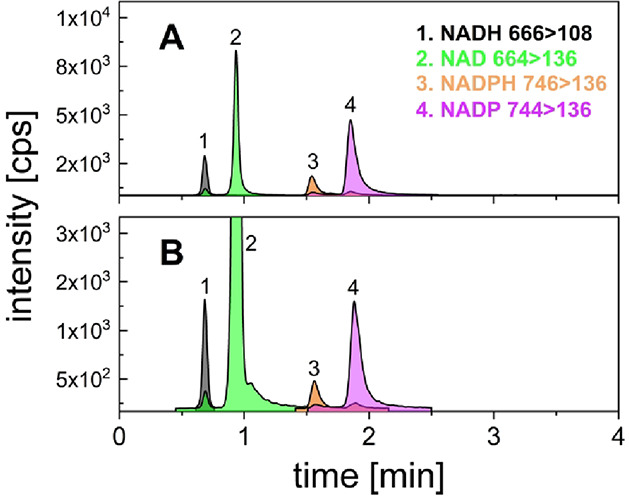
Extracted ion chromatograms of (A) a 1 μM standard mixture of NADH, NAD^+^, NADPH, and NADP^+^ and (B) a 10-fold diluted murine liver extract with 80% aqueous acetonitrile. Both analyses conducted on iHILIC-Fusion, SS, 30 × 2.1, 1.8 μm. Chromatogram B zoomed for better perceptibility of small peaks.

### Optimization of the HILIC LC-MS Method for Quantification of Nucleotides

First, MRM transitions were optimized by using direct injection of 10 μM individual nucleotides into MS and by using an Agilent Optimizer (Table S4). The two transitions with the highest intensity were used for further experiments. Initially, a mixture of 10 nucleotide standards and their derivates were used for method development. The concentration of each metabolite (c-GMP, AMP, CoA, ADP, GMP, ATP, GDP, UTP, CTP, GTP) was 10 μM. The initial method developed on a 15 cm column (iHILIC-(P) Classic, PEEK, 150 × 2.1, 5 μm) showed sufficient separation and peak shapes for all tested nucleotides within a 27 min analysis (data not shown). However, by using a shorter column (5 cm) and higher mobile phase flow rate (0.35 mL/min), it was possible to preserve the separation efficiency and resolution with a reduced time of analysis (15 min) (Figure 2). To develop a high-throughput targeted analysis, very short columns were also tested (2 and 3 cm). Even with optimized gradient elution, the 2 cm column lacked chromatographic resolution of some nucleotide pairs such as 8-oxo-dATP/dGTP, AMP/IMP, ADP/IDP, ATP/ITP, UMP/CMP, UDP/CDP, and UTP/CTP that are of similar structure and fragment similarly in collision-induced dissociation, thus demanding chromatographic separation. On the other hand, tuning of the flow rate and gradient on the 3 cm column resulted in a 6 min method that enables analysis of all 42 analytes contained in the standard mixture with acceptable peak resolution and 5 times faster (Figure 2). Both LC gradients were also tested with the 5 and 3 cm ACQUITY UPLC BEH Amide columns.

**Figure 2:**
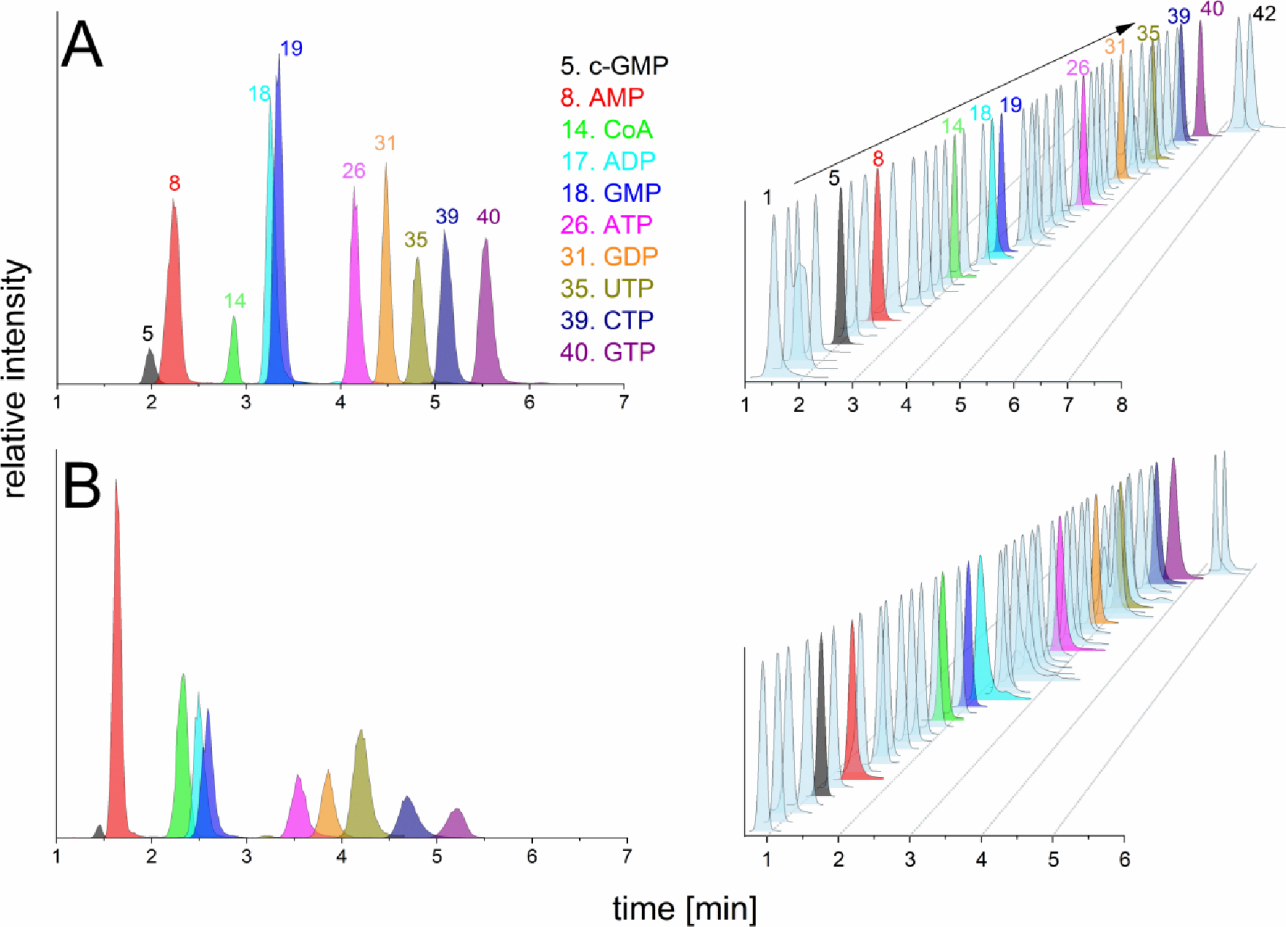
(A) Extracted ion chromatograms of 10 μM standard mixture of c-GMP, AMP, CoA, ADP, GMP, ATP, GDP, UTP, CTP, and GTP conducted on iHILIC-(P) Classic, PEEK, 50 × 2.1, 5 μm and analysis of 10 μM complex mixture of 42 phosphorylated standards. (B) Extracted ion chromatograms of 10 μM standard mixture of c-GMP, AMP, CoA, ADP, GMP, ATP, GDP, UTP, CTP, and GTP performed on iHILIC-(P) Classic, PEEK, 30 × 2.1, 5 μm and analysis of 10 μM complex mixture of 42 phosphorylated standards. The elution order of all nucleotide standards for both columns is listed in Table S4.

These columns, with nearly identical parameters, enabled separation of standard nucleotide mixtures similar to that of iHILIC-(P) Classic (Figure S3). However, due to worse peak resolution of the critical pairs of metabolites such as UTP and CTP, they were excluded from further testing, and subsequent experiments were conducted only on iHILIC-(P) Classic.

### Method Validation for Quantification of NAD Metabolites

The method for quantification of NAD metabolites was most sensitive for detection of NADH and NAD^+^ with an LOQ of 1 nM, whereas NADPH was detected with the lowest sensitivity with an LOQ of 25 nM (Table S3). The LOQ values were considered the starting points for calibration, which ranged from the LOQ up to 10 μM and sufficed for quantification of all NAD metabolites in the 10 mg liver samples. Accuracy in liver extracts was evaluated through spiking into the liver extracts at three concentration levels, and the accuracy was in an acceptable range of 100 ± 15% at all levels. Precision and repeatability of the method, expressed as RSD, did not exceed 5%. Carry-over in % of peak areas in blanks injected after the highest calibration point (10 μM) showed that there were no significant carry-over effects with peak areas in blanks below 1% for all metabolites; NADH (0.05%) and NADP^+^ (0.9%) were the analytes with the lowest and highest carry-over, respectively. However, there was no carry-over detected from the liver extracts as the concentrations in real samples did not reach the critical value for the carry-over to be significant; for example, NADP^+^ was detected at concentration levels between 400 and 500 nM on average. The endogenous matrix of the samples resulted in minor ion suppression (within 8%) of all tested metabolites except for NAD^+^, whose signal was increased by 6% through the ion enhancement effect. The matrix effects were compensated for by using isotopically labeled standards, and therefore the accuracy of the method was not compromised. The stability of the NAD^+^ cofactors in extracts stored in the autosampler for 24 h was within 100 ± 3% of the signal measured in the fresh extracts. The chemical, pH-dependent stability was evaluated on individual standards dissolved in different solvents, as described in Materials and Methods, and stored over the course of 46 h. The peak areas of NAD metabolites were monitored under different pH conditions, as well as peak areas of the degradation products – NAM, AMP, ADP, ATP, ADPR, and NMN – that were analyzed with the same method (Figure S4 and Table S5). It has been reported many times that the oxidized forms (NAD, NADP^+^) are stable in acidic solvents while the reduced forms withstand extractions with alkaline solvents.^33,34^ Our results suggest that NAD^+^ and NADP^+^ remain stable under acidic conditions (0.6 M perchloric acid and 0.1 M formic acid) and that they degrade at a fast rate under alkaline conditions (0.1 M sodium hydroxide), which agrees with the literature. The reduced metabolites proved to degrade readily in strong acid (0.6 M perchloric acid), where significant degradation occurred already in the fresh solvent (after 20 min from preparation), whereas they were relatively stable in 0.1 M formic acid within 2 h from preparation. Strikingly, NADH and NADPH showed poor stability in alkaline conditions (0.1 M sodium hydroxide) with their degradation to 11 and 17% of their original amount after 46 h, respectively (Figure S5). Therefore, it is essential to neutralize the sample extracts shortly after addition of 0.1 M formic acid during the sample preparation. The analysis of degradation products of NADP^+^ and NADPH provided peaks with MRM transitions of ADP and ATP but differed in the retention times. Presumably, these degradation products corresponded to adenosine-2’-5′-diphosphate (ADP-2’-5′) and adenosine-2’-5′-diphosphate (ATP-2’-5′) – the isomeric analogues of ADP and ATP.

### Method Validation for Quantification of Nucleotides

Quantification methods for nucleotides and their derivatives achieved LOQs of 5–30 nM for most analytes using both 15 and 6 min methods. Recovery was determined by spiking experiments with human plasma, and most nucleotides achieved recovery over 80% on both columns tested. A few nucleotides reached recoveries only around 50%, for example, GTP, 8-oxo-dGTP, 8-oxo-dATP, Suc-CoA, CoA, and 3-dp-CoA were recovered only at 8%, most likely due to interactions of these metabolites with proteins found in the human plasma (Figure S6). Accuracy, assessed in human plasma and the same samples spiked with standards at two concentration levels, was within 100 ± 15% for most analytes, except for those without isotopically labeled standards (Table S6, S7). External calibration was used for these analytes, and therefore the accuracy was compromised by the sample matrix resulting in accuracy values out of the validation criteria (100 ± 15%). The accuracy could be improved by using isotopically labeled internal standards for metabolites like AICAR, c-di-GMP, c-GMP, cyclocreatine, dUMP, dUTP, IDP, and P-creatine. The bacterial alarmones—ppGpp and pppGpp—in mupirocin-treated bacteria were quantified based on the standard addition method through spiking the extracts by (i) 1 μM, (ii) 5 μM, and (iii) 10 μM standards of ppGpp and pppGpp. This approach ensured reliable quantification despite the absence of internal standards.

For a 15 min gradient on a 5 cm column, variability in precision did not exceed 10% in most cases, and repeatability of peak areas remained below 15%. In the case of the 6 min method on a shorter column, variability in precision and variation in repeatability did not exceed 5% for most analytes (Table S7). The analysis of blank samples measured immediately after real samples in sequence confirmed the absence of undesirable carry-over effects for both methods. The matrix effects observed on shorter column were comparable with 5 cm column in the late-eluting compounds; however, matrix effects differ significantly in the early eluting compounds, for example, c-GMP, dUMP, c-di-AMP, AMP, AICAR, IMP, dGMP, ADP, and GMP. Cyclocreatine was the only metabolite whose signal was significantly improved via ion enhancement effect on both columns in spiked human plasma samples (Figure S7). Despite the strong matrix effects in human plasma, biologically relevant metabolites were quantified in all tested tissues. Consequently, a possible elimination of the matrix effects by SPE or LLE was tested on samples of human plasma spiked with nucleotide standards.

### Quantification of NAD Metabolites in Murine Liver, Skeletal Muscle, and White Adipose Tissue

To reliably quantify NAD metabolites by LC-MS/MS, a suitable extraction solvent is needed for fast enzyme quenching and efficient extraction, and suitable internal standards should also be used to account for extraction recovery and matrix effects. The extraction of NAD^+^, NADP^+^, NADH, and NADPH was previously tested and optimized not only in regard to extraction efficiency but also to interconversion between reduced and oxidized forms.^20^ It was shown that 0.1 M formic acid in 80% aqueous organic solvent with the following neutralization offers the best option for extraction of NAD metabolites from tissues in terms of low interconversion and high accuracy of quantification. Therefore, 0.1 M formic acid in 80% aqueous methanol followed by neutralization with 9% aqueous ammonium bicarbonate was used in this study. The extracts were diluted 10-fold with 80% aqueous acetonitrile before the analysis by LC-MS/MS. The absolute quantification was conducted by means of a 14-point internal calibration with isotopically labeled standards (NAD-D_4_, NADH-D_4_) that were spiked in the samples before extraction. Despite the unavailability of isotopically labeled standards for NADP^+^ and NADPH, the accuracy measured in the liver extracts was acceptable even when using NAD-D_4_ and NADH-D_4_ for quantification of NADP^+^ and NADPH, respectively (Table S3). The reference values in the literature for NAD metabolites differ substantially depending on the biological variability, extraction solvent, and/or on the type of quantification approach.^20,35^ The recent metadata analysis of the reported values obtained by multiple methods^36^ showed high variability in the quantified amounts of the NAD metabolites in murine liver (Table S8). Nevertheless, our LC-MS/MS quantitation resulted in similar values obtained with comparable extraction and quantification approach.^37^ The concentrations of NAD^+^, NADH, NADP^+^, and NADPH were also determined using a previously published enzymatic cycling assay.^38,39^ Moreover, the NAD cofactors were quantified in murine skeletal muscle and adipose tissue, with all values listed in Table S8.

### Quantification of Nucleotides in Murine Liver, Skeletal Muscle, Adipose Tissue, Bacterial Extracts, and Human Plasma

Overall, analyses performed on a 5 cm column with a 15 min gradient exhibited higher signals for most monitored analytes than the 6 min method on a 3 cm column providing similar results in terms of metabolite identification and concentration levels (Figure 3).

**Figure 3:**
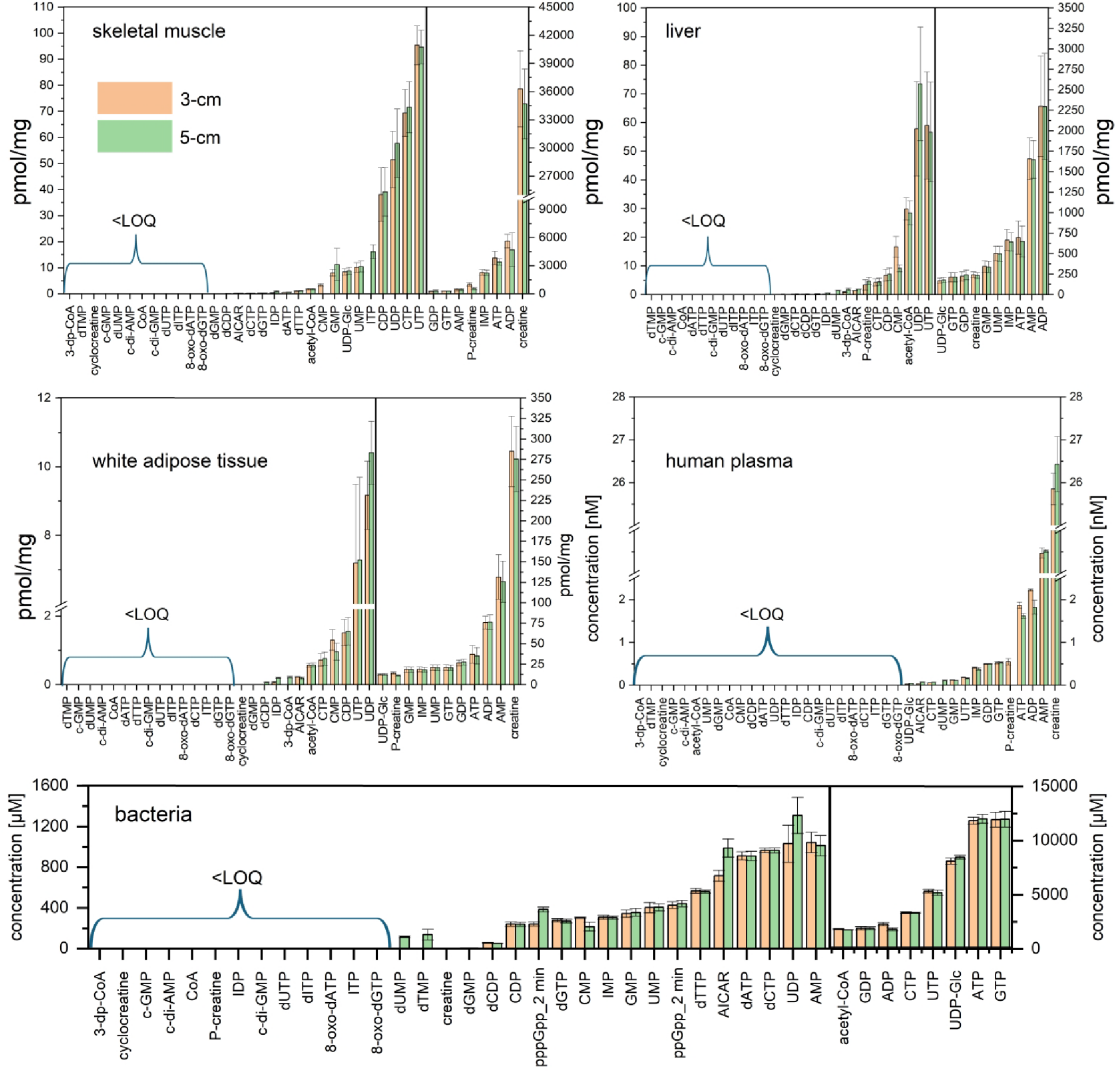
Concentration [pmol/mg] of nucleotides and their derivates identified in murine liver, white adipose tissue, and skeletal muscle tissue, as well as concentration of nucleotides [nM] in human plasma (*n* = 5) and bacteria (*E. coli*) extracts (untreated, *n* = 3, μM per total cell volume). Concentrations of ppGpp and pppGpp from 2 min treatment are included for column comparison. The samples were analyzed by an optimized LC-MS method using iHILIC-(P) Classic, PEEK, 50 × 2.1 mm, 5 μm column and 15 min gradient and compared to a method on an iHILIC-(P) Classic, PEEK, 30 × 2.1 mm, 5 μm column and 6 min gradient. For exact concentrations, see Table S9. The NAD cofactors were analyzed with a different method, and their comparisons are located in Table S8.

The data revealed distinct metabolite concentration patterns across different types of samples–tissues, bacteria, or plasma, reflecting their unique metabolic roles. The extraction with acidified 80% methanol that was subsequently neutralized with ammonium bicarbonate revealed high levels of nucleotide-related metabolites such as ATP, ADP, and AMP in the liver, underscoring its central role in energy metabolism and biosynthesis. Muscle tissue showed high concentration of creatine, ATP, ADP, AMP, P-creatine, GDP, and GTP. The same method was applied to a study,^40^ where we quantified mal-CoA levels with the method on the 3 cm column in gastrocnemius and quadriceps muscles in mice and the method proved to be significantly robust to clearly detect genotype-specific levels of mal-CoA in pantothenate kinase 4-deficient mice compared to the control. Adipose tissue contains lower metabolite concentrations, reflecting its primary function in storing energy rather than active metabolism, with creatine, AMP, ADP, and ATP being the most abundant metabolites. Plasma shows relatively low levels of metabolites, probably because its main role is a transport medium, so its metabolite levels are expected to be lower than those of tissues. The HILIC methods offered the best coverage of polar metabolites in bacteria (*E. coli*) extracted with 1 M acetic acid, where 28 phosphorylated metabolites were quantified including ppGpp and pppGpp in bacteria treated with mupirocin (a cell number for *E. coli* growing in LB media was estimated at 3.9 × 10^8^ and the cell volume at 1 × 10^–15^ L). Treatment of the bacteria with isoleucyl-tRNA synthetase (IleRS) inhibitor mupirocin (also known as pseudomonic acid) induced the so-called stringent response, that is, overproduction of alarmone nucleotides ppGpp and pppGpp by the stringent factor RelA in response to accumulation of deacylated tRNA^Ile^.^41^ Bacterial samples exhibit rapid and distinct metabolic turnover, with significant fluctuations in metabolites such as IMP, AICAR, dUMP, GMP, CMP, ppGpp, pppGpp, dTTP, dATP, GDP, CTP, UTP, GTP, and UDP-Glc between untreated and mupirocin-treated samples (Figure 4). The most significant changes were detected in decreasing concentrations of AICAR and IMP as the intermediates in purine synthesis, thus precursors for synthesis of GDP and GTP that are subsequently phosphorylated to ppGpp and pppGpp.^100^ The changes of other nucleotides reflect adaptation of bacteria’s metabolism to the stringent response. For example, synthesis of polysaccharides as building blocks of the bacterial membranes might be downregulated, and thus UDP-Glc accumulates as it is the precursor for carbohydrate synthesis.^101^

**Figure 4:**
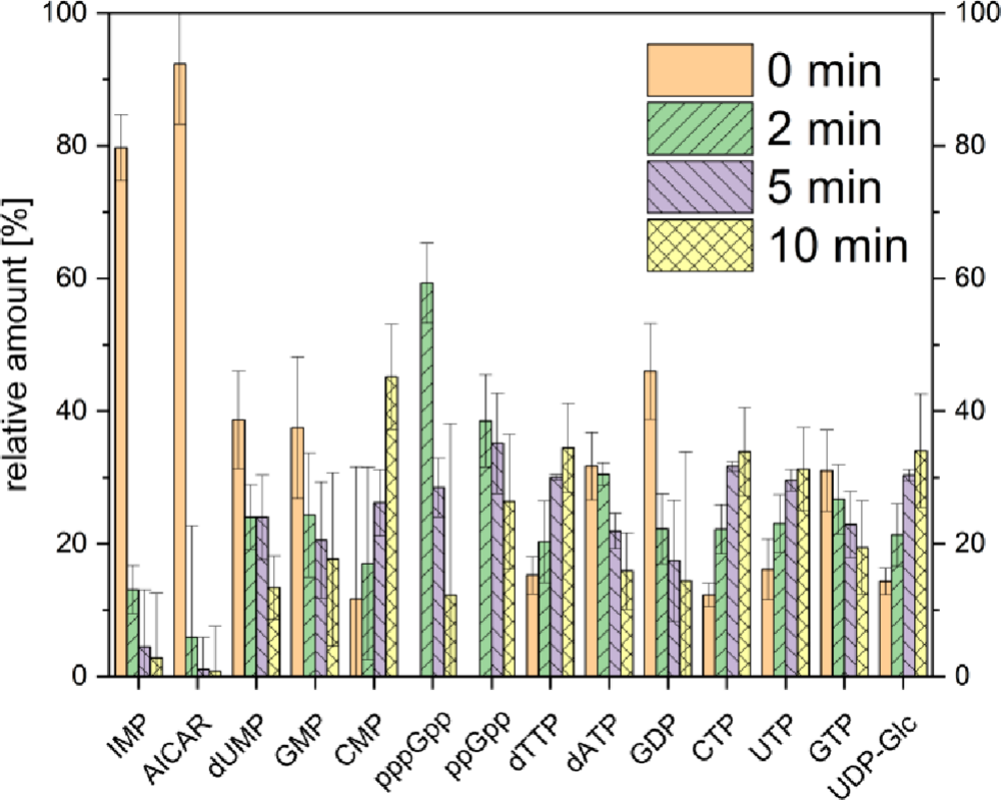
Bacterial nucleotides with the most significant mupirocin-induced changes after 0, 2, 5, and 10 min of treatment measured with iHILIC-(P) Classic, PEEK, 50 × 2.1 mm, 5 μm column. For exact concentrations of all quantified nucleotides in bacteria, see Table S10.

### ^13^C-Labeling in Mouse Pluripotent Stem Cells Treated with ^13^C_6_-Glc

As a proof-of-concept experiment, the cell extracts were analyzed with both method—for labeling in NAD cofactors as well as in other nucleotides—with high-resolution MS (HRMS). Because only NADH and NAD were detected with HRMS, the labeling was also determined on a more sensitive triple quadrupole instrument in MRM mode to detect NADP and NADPH. In triple quadrupole experiment, a fragment of 136 (unlabeled adenine) was used as a product ion and the labeling correlates well with enrichment obtained from HRMS even on the other isotopologues other than *m* + 5 and *m* + 10, suggesting minor labeling in adenine after 5-h ^13^C-Glucose treatment (Figure S8). For other nucleotides, the longer, 15 min gradient on a 5 cm column was used together with HRMS. Following this, 10 compounds were reliably detected—creatine, AMP, CoA, UDP, UDP-Glc, ATP, GDP, UTP, CTP, and GTP. In all detected metabolites except for creatine, *m* + 5 was the predominant isotopologue with abundance from 22% in UDP-Glc to 54% in GDP, suggesting that the ribose unit in the structures is labeled with five ^13^C atoms through the pentose phosphate pathway. Metabolites like NAD cofactors and UDP-Glc had one more abundant isotopologue *m* + 10 and *m* + 11 cluster, respectively since they in addition contain either 2 ribose units (NAD) or one ribose and one glucose unit (UDP-Glc).

### Stability Testing of Nucleoside Triphosphates

Method optimization requires the frequent use of standards and stock solutions, which must be monitored for stability to avoid degradation. Few stability studies exist, but nucleotides are known to be unstable in acidic conditions^42^ and stable for months at 4 °C or years at −20 °C.^43^ However, stability during freeze–thaw cycles remains unstudied, raising concerns about solvent effects and degradation during storage and repeated freezing. Testing the stability of nucleotides subjected to repeated freeze–thaw cycles revealed no significant degradation of nucleotides in all of the tested solvents. Peak areas for all four nucleoside triphosphates and their corresponding mono- and diphosphates were monitored with dual detection—UV/vis and MS/MS. Significant analyte degradation was not observed even after 14 freeze–thaw cycles (Table S11). Similarly, no significant degradation was observed for standards stored for 3 days in autosampler with stable temperature of 5 °C.

### Matrix Effects in HILIC-LC-MS/MS

In biological samples such as plasma or tissue extracts, lipids and other interfering background matrices are present in high concentrations, causing undesired matrix effects in HILIC analysis. Solid-phase extraction (SPE) and liquid–liquid extraction (LLE) are widely used sample preparation methods in various omics fields.^10,44–46^ During method validation, strong matrix effects were observed across all of the sample types. Therefore, LLE and SPE were tested to purify the samples and enhance nucleotide signals. Both methods were optimized to maximize lipid removal, as lipids are easily separated based on higher hydrophobicity while preserving nucleotides for HILIC analysis. Initial testing identified ethyl acetate and water as the most suitable, user- and environmentally friendly solvents compared to dichloromethane, chloroform, and chloroform/methanol (Folch extraction).^47^ To test the procedure, a mix of six lipid standards from different classes was used: triglyceride (TG), phosphatidylcholine (PC), phosphatidylglycerol (PG), phosphatidylinositol (PI), phosphatidylethanolamine (PE), and sphingomyelin (SM). Acidifying the aqueous phase before LLE proved to be essential for transferring lipids into the organic phase. At higher pH, lipids remained in the aqueous phase with nucleotides, likely due to ion pairing,^48,49^ which acidification prevents. The use of 1% formic acid significantly improved lipid transfer into the organic layer. The method was applied to 50 μL of a human plasma extract spiked with nucleotide standards (10 μM). The extract was diluted 10× with water and applied to a two-phase ethyl acetate/water (1:1, v/v) system with 1% formic acid. LLE with ethyl acetate resulted in ∼50% separation of monitored lipids from nucleotides, with the nucleotide loss in the aqueous phase limited to 2%.

During optimization of the SPE procedure, two commercially available column types were tested: (i) ISOLUTE C18 and (ii) iSPE-HILIC. The C18 phase experiment followed a five-step reversed-phase elution protocol. First, a 10× diluted plasma extract with water was applied to the column preconditioned with acetonitrile and 0.1% TFA in 5% acetonitrile. This step aimed to retain hydrophobic lipids on the sorbent, while hydrophilic nucleotides passed in the flow-through fraction. The next two steps involved washing with 0.1% TFA in 5% acetonitrile to remove the remaining hydrophilic residues. The final two steps eluted the retained lipids using a 1:1 mixture of isopropyl alcohol and methanol. Most of the nucleotides eluted in the flow-through or in the first acetonitrile fraction. Lipidomic analysis across all five fractions confirmed that the C18 SPE procedure efficiently extracted nearly 100% decanoylcarnitine and 90% lysophospholipid 18:1. Other monitored lipids were primarily eluted in the isopropyl alcohol fraction (up to ∼30%), while the remaining 70% eluted with nucleotides in the flow-through.

The HILIC-SPE protocol was tested by using iSPE-HILIC cartridges. This method was adapted from the literature^50^ and optimized using experience from HILIC chromatography. Initially, the spiked plasma extract (5× diluted with 90% methanol) was loaded onto the column, with hydrophobic lipids eluting in the flow-through fraction. Acetonitrile was used in a second step to remove residual lipids, while nucleotides were finally eluted using water. The protocol retained nearly 100% of all monitored lipids in the flow-through and acetonitrile fractions.

The recovery was calculated for all three sample preparation techniques on spiked human plasma extracts, suggesting that LLE offers the best recovery of nucleotides with overall recovery close to 100% except for 3-dp-CoA, P-creatine, and CoA. A simple extract cleanup on C18-SPE provided recoveries of only over 60% for most nucleotides, and the HILIC-SPE performed the worst in terms of recovery, especially for late-eluting nucleotide triphosphates. Despite the low overall recovery on HILIC-SPE, the signal was significantly enhanced for some nucleotides like dUMP, IMP, dGMP, CMP, GMP, and UDP-Glc, suggesting that the HILIC-SPE efficiently eliminates matrix from human plasma coeluting with these metabolites (Figure S9).

## Conclusions

In this study, we developed and optimized HILIC-LC-MS-based methods for the analysis of phosphorylated metabolites in complex biological samples such as bacteria, human plasma, and murine muscle, adipose, and liver tissues. At first, a 15 min analysis using a 5 cm column was developed for analysis of nucleotides, deoxynucleotides, and coenzymes. By optimizing the column length and elution gradient, the analysis time was reduced to 6 min on a 3 cm column while retaining sufficient separation efficiency. As separation of NAD metabolites (NAD^+^, NADH, NADP^+^, NADPH) on iHILIC-(P) Classic columns was insufficient, a specific HILIC method for analysis of NAD metabolites was developed by using a 3 cm iHILIC-Fusion column. This method allowed baseline separation of the NAD metabolites within 4 min and enabled their absolute quantification in murine liver, skeletal muscle, and white adipose tissue.

All of the HILIC methods were validated in terms of sensitivity, linearity, accuracy, matrix effects, recovery, and precision. The overall sensitivity (LOQ) for most of the tested compounds was in the range 5–30 nM with CTP having the lowest LOQ (3 nM) and ITP having the highest LOQ values (70 nM). The long-term stability of nucleotides was tested on a mixture of ATP, GTP, CTP, and UTP in 50% aqueous methanol, 50% aqueous acetonitrile, and in water stored at −20 °C and showed no significant degradations of tested compounds over 14 freeze–thaw cycles. The validated HILIC methods enabled the absolute quantification of phosphorylated metabolites in complex biological samples by using isotopically labeled standards for most analytes. We also showed that the methods are applicable to ^13^C labeling studies with the possibility to trace phosphorylated compounds.

Eventually, to reduce matrix effects, the test samples of human plasma spiked with nucleotides were subjected to liquid–liquid extraction and solid-phase extraction. The LLE and C18-SPE reached satisfactory recovery but failed to eliminate the sample matrix in human plasma extracts. Meanwhile, the HILIC-SPE approach lacked sufficient recovery especially for nucleotide triphosphates; however, it enhanced signal of some nucleotides such as IMP, dUMP, dGMP, CMP, GMP, and UDP-Glc.

## Supplementary Material


