# Supplementary material for: Rapid Analysis of NAD and Other Phosphorylated Metabolites in Complex Biological Samples by Hydrophilic Interaction Liquid Chromatography Coupled with Tandem Mass Spectrometry
Source: Anal Chem. 2026 Apr 8;98(15):11428–37. doi: 10.1021/acs.analchem.6c00721 (PMC13103929; doi:10.1021/acs.analchem.6c00721)
Supplement: Supplementary file 1 [file ac6c00721_si_001.pdf]

Supplementary information

Rapid analysis of NAD and other phosphorylated metabolites in complex biological samples by HILIC-MS/MS

Adela Pravdova[1,2,3,4], Maximilian Kleinert[5,6], John Henderson[7], Eleni Kafkia[8], David Pladevall-Morera[8], Caio Y. Yonamine[7], Jonas T. Treebak[7], Tetiana Brodiazhenko[9], Ilya Terenin[10], Jan Jakub Zylicz[8], Thomas Moritz[7], Ondrej Hodek[1,4]*

[1]Department of Forest Genetics and Plant Physiology, Swedish University of Agricultural Sciences, Umeå, Sweden

[2]Institute of Organic Chemistry and Biochemistry of CAS, Prague, Czech Republic

[3]Department of Analytical Chemistry, Faculty of Science, Charles University, Prague, Czech Republic

[4]Swedish Metabolomics Centre, Umeå, Sweden

[5]Department of Molecular Physiology of Exercise and Nutrition, German Institute of Human Nutrition (DIfE), Potsdam-Rehbruecke, 14558 Nuthetal, Germany

[6]German Center for Diabetes Research (DZD), 85764 Munich, Germany

[7]Novo Nordisk Foundation Center for Basic Metabolic Research, Faculty of Health and Medical Sciences, University of Copenhagen, Blegdamsvej 3B, 2200 Copenhagen N, Denmark

[8]Novo Nordisk Foundation Center for Stem Cell Medicine, reNEW, University of Copenhagen, Copenhagen, Denmark

[9]Icosagen Cell Factory OÜ, Tartu, Estonia

[10]Lund University, SciLifeLab, Lund, Sweden

Table of Contents

Table S1 Chemicals and reagents.

Preparation of standard solutions.

Sample preparation of human plasma.

Sample preparation of murine liver, adipose and skeletal muscle tissue.

Sample preparation of bacterial extracts.

Culture of mouse pluripotent stem cells.LC-MS method for lipid analysis.

Method validation for separation of NAD metabolite.

Method validation for separation of nucleotides and their derivates.

Stability testing of standard nucleotides.

Table S2 Description of the scoring system for calculation of the quality score.

Figure S1 Quality score calculated from signal-to-noise ratio and tailing factor of a mixture of standards analyzed on 5 tested columns under various pH of buffer in the mobile phase.

Table S3 LC-MS parameters for NAD metabolites with respective validation parameters such as limits of detection, limits of quantification, linear calibration range with coefficient of determination, precision, repeatability in peak areas, and matrix effects.

Figure S2 Comparison of separation on (**1**) iHILIC-(P) Classic, PEEK, 50×2.1, 5 µm and (**2**) iHILIC-Fusion, SS, 50×2.1, 1.8 µm.

Table S4 LC-MS/MS parameters for nucleotides and their derivates.

Figure S3 Extracted ion chromatograms of 10 µM standard mixture of c-GMP, AMP, CoA, ADP, GMP, ATP, GDP, UTP, CTP and GTP conducted on (A) ACQUITY UPLC BEH Amide, 50×2.1 mm, 1.7 µm, 130Å and (B) ACQUITY UPLC BEH Amide, 30×2.1 mm, 1.7µm, 130Å.

Figure S4 Extracted ion chromatogram of 10 µM standard mixture of NAD metabolites and their degradation products.

Table S5 Degradation of individual NAD metabolites in acidic, alkaline, and neutral conditions with respective degradation products.

Figure S5 Stability of NAD metabolite standards dissolved in 0.6 M perchloric acid, 0.1 M sodium hydroxide, and 0.1 M formic acid in 80% methanol**.**

Figure S6 Recovery determined on spiked human plasma, analyzed on iHILIC - (P) Classic 50 × 2.1 mm, 5 µm and iHILIC - (P) Classic 30 × 2.1 mm, 5 µm.

Table S6 Validation parameters for nucleotides analyzed on iHILIC-(P) Classic, PEEK, 50×2.1, 5 µm with respective limits of quantification, linear calibration range, precision, accuracy, and repeatability of peak areas.

Table S7 Validation parameters for nucleotides analyzed on iHILIC - (P) Classic 30 x 2.1 mm, 5 µm with respective limits of quantification, linear calibration range, precision, accuracy, and repeatability of peak areas.

Figure S7 Comparison of matrix effects in spiked human plasma measured on iHILIC - (P) Classic 50 × 2.1 mm, 5 µm and iHILIC - (P) Classic 30 x 2.1 mm, 5 µm.

Table S8 Concentration of NAD metabolites in murine liver, murine skeletal muscle, and murine white adipose tissue, median ± confidence interval at a confidence level α = 0.95 with RSD in % in parentheses.

Table S9 Concentrations of nucleotides and other phosphorylated metabolites in murine tissue, human plasma and E. coli quantified on 3-cm and 5-cm iHILIC-(P) Classic, PEEK, 50×2.1, 5 µm.

Table S10 Concentrations of nucleotides and other phosphorylated metabolites in E. coli treated with mupirocin for 0, 2,5, and 10 minutes. The metabolites were quantified on 3-cm and 5-cm iHILIC-(P) Classic, PEEK, 50×2.1, 5 µm.

Table S11 Distribution (%) of mono-, di-, and triphosphates in standard nucleotide samples dissolved in 50% aqueous methanol, water or 50 % aqueous acetonitrile stored in -20 °C, after multiple freeze-thaw cycles, measured by LC-MS.

Figure S8 13C-labelling detected in NAD cofactors in mouse pluripotent stem cells

Figure S9 Recovery and gain factor calculated from spiked plasma samples analyzed by iHILIC - (P) Classic 50 × 2.1 mm, 5 µm.

References

Table S1 Chemicals and reagents.

Chemical name	Abbreviation	Vendor
Acetonitrile (LC–MS grade)	ACN	Sigma–Aldrich (MO, USA)
2-propanol	IPA	
Methanol	MeOH	
Ammonium acetate	AmAc	
Formic acid	FA	
Uridine 5′- (trihydrogen diphosphate) sodium salt	UDP	
1,2-Diacyl-*sn*-glycero-3-phosphocholine	PC	
N-Acyl-4-sphingenyl-1-O-phosphorylcholine	SM	
1,2-Diacyl-*sn*-glycero-3-phospho-(1-*rac*-glycerol)	PG	

2'-Deoxycytidine 5'-diphosphate sodium salt hydrate	dCDP	Cayman Chemical (MI, USA)
2,2,2-Trifluoroacetic acid	TFA	Merck-Millipore (MA, USA)
MilliQ deionized water	H_2O	
Adenosine 5′-monophosphate monohydrate	AMP	
Adenosine 5′-diphosphate sodium salt	ADP	
Nicotinamide	NAM	
β-nicotinamide mononucleotide	NMN	
Guanosine 5′-monophosphate disodium salt hydrate	GMP	
Uridine 5′-diphosphoglucose disodium salt hydrate	UDP-Glc	
Inosine 5′-triphosphate trisodium salt	ITP	
Inosine-5'-monophosphate Disodium Salt octahydrate	IMP	
Guanosine 5′-diphosphate sodium salt	GDP	
Cytidine 5′-monophosphate disodium salt	CMP	
Acetyl coenzyme A trisodium salt	Ac-CoA	
Succinyl coenzyme A sodium salt	Suc-CoA	
Malonyl coenzyme A lithium salt	Mal-CoA	
2′-deoxy-uridine-5′-triphosphate	dUTP	
Creatine phosphate	P-creatine	
Thymidine 5′-triphosphate sodium salt solution	dTTP	
Adenosine-5′-diphosphoglucose disodium salt	ADP-Glc	
AICA-Riboside, 5′-Phosphate	AICAR	
Guanosine-3′,5′-cyclic monophosphate calcium salt	c-GMP	
Malonyl-$^{13}C_3$ coenzyme A lithium salt	mal-CoA-$^{13}C_3$	
Adenosine-$^{15}N_5$ 5′-diphosphate disodium salt	ADP-$^{15}N_5$	
Acetyl-1,2-$^{13}C_2$ coenzyme A lithium salt	Ac-CoA-$^{13}C_2$	
Uridine-$^{13}C_9$ $^{15}N_2$ 5′-triphosphate disodium salt solution	UTP-^{13}C10 $^{15}N_2$	
2′-Deoxyguanosine-$^{13}C_{10}$ $^{15}N_5$ 5′-monophosphate disodium salt	dGMP-^{13}C10 $^{15}N_5$	
Guanosine-$^{13}C_{10}$ 5′-triphosphate disodium salt	GTP-$^{13}C_{10}$	
Cytidine-$^{15}N_3$ 5′-triphosphate disodium salt	CTP-$^{15}N_3$	
Adenosine-$^{13}C_{10}$ $^{15}N_5$ 5′-triphosphate disodium salt	ATP-$^{13}C_{10}$ $^{15}N_5$	
Adenosine-$^{13}C_{10}$ $^{15}N_5$ 5′-monophosphate disodium salt	AMP-$^{13}C_{10}$ $^{15}N_5$	
2′-Deoxyguanosine-$^{13}C_{10}$ $^{15}N_5$ 5′-triphosphate disodium salt	dGTP-$^{13}C_{10}$ $^{15}N_5$	
2′-Deoxyadenosine-$^{13}C_{10}$ $^{15}N_5$ 5′-triphosphate disodium salt	dATP-$^{13}C_{10}$ $^{15}N_5$	
2′-Deoxycytidine-$^{13}C_9$ $^{15}N_3$ 5′-triphosphate disodium salt	dCTP-13C_{10} $^{15}N_5$	
Guanosine-$^{15}N_5$ 5′-monophosphate disodium salt	GMP-$^{15}N_5$	
Cytidine-$^{13}C_9$ $^{15}N_3$ 5'- diphosphate lithium salt solution	CDP-$^{13}C_9$ $^{15}N_3$	Silantes (Germany)
Uridine-$^{13}C_9$ 5'- monophosphate lithium salt	UMP-$^{13}C_9$	
Inosine-$^{15}N_4$ 5'- monophosphate lithium salt	IMP-$^{15}N_4$	
Thymidine-$^{13}C_{10}$ 5'- triphosphate lithium salt	dTTP-$^{13}C_{10}$	
Guanosine-$^{13}C_{10}$ $^{15}N_5$ 5'- diphosphate lithium salt	GDP-$^{13}C_{10}$ $^{15}N_5$	
Cytidine-$^{13}C_9$ 5'- monophosphate lithium salt	CMP-$^{13}C_{10}$	
Deoxycytidine-$^{15}N_3$ 5'- diphosphate lithium salt	dCDP-$^{15}N_3$	
Inosine-$^{13}C_{10}$ $^{15}N_4$ 5'- triphosphate lithium salt	ITP-$^{13}C_{10}$ $^{15}N_4$	
Uridine-$^{13}C_9$ $^{15}N_2$ 5'- triphosphate lithium salt	UDP-$^{13}C_9$ $^{15}N_2$	
8-Oxo-2'-deoxyguanosine-5'-triphosphate sodium salt	8-oxo-dGTP	Jena Bioscience (Germany)
8-Oxo-2'-deoxyadenosine-5'-triphosphate sodium salt	8-oxo-dATP	
Inosine 5'-diphosphate, disodium salt	IDP	Santa Cruz Biotechnology (TX, USA)
Coenzyme A sodium salt hydrate	CoA	Glentham Life Science (Germany)
Adenosine 5′-diphosphoribose sodium salt	ADPR	MedChemExpress (NJ, USA)
β-nicotinamide adenine dinucleotide hydrate	NAD	Roche Diagnostics (Switzerland)
β-nicotinamide adenine dinucleotide phosphate disodium salt	NADP	
β-nicotinamide adenine dinucleotide, reduced disodium salt hydrate	NADH	

β-nicotinamide adenine dinucleotide 2′-phosphate reduced tetrasodium salt hydrate	NADPH	
adenosine 5′-triphosphate<br>2'-Deoxyguanosine-5'-monophosphate disodium salt hydrate<br>Cytidine-5'-diphosphate disodium salt<br>2'-Deoxyuridine-5'-monophosphate disodium salt<br>2'-deoxyinosine 5'-triphosphate<br>2'-deoxyguanosine 5'-triphosphate<br>Uridine 5'-triphosphate<br>Guanosine 5'-triphosphate<br>Cytidine 5'-triphosphate<br>2'-deoxyadenosine 5'-triphosphate	ATP<br>dGMP<br>CDP<br>dUMP<br>dITP<br>dGTP<br>UTP<br>GTP<br>CTP<br>dATP	Thermo Fisher Scientific (MA, USA)
2'-Deoxythymidine-5'-monophosphate disodium salt	dTMP	Chem-Impex International (IL, USA)
Chloroform	$CHCl_3$	Honeywell (NC, USA)
Ethyl Acetate	EtAc	J.T.Baker (NJ, USA)
β-nicotinamide adenine dinucleotide-D_4<br>β-nicotinamide adenine dinucleotide, reduced diammonium salt-D_4	NAD-D_4<br>NADH-D_4	Toronto Research Chemicals (ON, Canada)
Creatine METHYL-D_3<br>Nicotinamide adenine dinucleotide ammonium salt RIBOSE-$^{13}C_5$<br>Uridine diphosphate-α-D-glucose disodium salt	creatine-D_3<br>NAD-$^{13}C_5$<br>UDP-Glc-$^{13}C_6$	Cambridge Isotope Laboratories (UK)
Guanosine pentaphosphate<br>Guanosine tetraphosphate	pppGpp<br>ppGpp	prepared as described [1]

Preparation of standard solutions. Stock solutions of nucleotides were prepared at a 10 mM concentration in 10 mM Tris-HCl buffer of pH 7. The following dilutions to 1 mM were conducted with 50% methanol/water (*v*/*v*). Stock solutions of lipids were prepared by dissolving lyophilized powder in isopropyl alcohol/methanol 1/1 (*v*/*v*) at a concentration of 1 mg/mL. Stock solutions of acyl-coenzymes A (Ac-CoA, Suc-CoA, Mal-CoA) were prepared in 50 mM ammonium acetate and 10 mM ascorbic acid in glass vials. The working solutions of acyl-CoA were diluted with 50% aqueous methanol containing 1 mM ascorbic acid.

Sample preparation of human plasma. The human plasma samples were obtained from Biobanken norr (Laboratory Medicine within Region Västerbotten). The informed consent from all human subjects was obtained before submitting their samples into the Biobank. All experiments were performed in accordance with relevant guidelines and regulations. The plasma samples consisted of a pooled plasma from adult male and female donors. The 20-µL aliquots of human plasma were spiked with a mix of isotopically labeled standards and extracted with 180 µL 80% methanol with 0.1 M formic acid. After 5 s of vortexing,

20 μL of 9% ammonium bicarbonate was added, and the sample was vortexed for 5 s. For protein precipitation, the samples were incubated at −20 °C for 2 hours. Then, the samples were centrifuged at 14,000 *g* for 10 min. The supernatants were transferred into LC vials and evaporated in miVac Quattro Concentrator (Genevac, UK). Prior to the LC-MS analysis, the samples were reconstituted in 50 μL of 50% aqueous methanol. The human plasma samples used for evaluation of matrix effects samples were obtained from Biobank based on anonymized basal plasma samples from a previous study[2]. The informed consent from all human subjects was obtained before submitting their samples into the Biobank. All experiments were performed in accordance with relevant guidelines and regulations including approval by the ethics committee of Southern Denmark (S-20210001) and the Copenhagen region (H-21015422) and registered in a public database, Clinicaltrials.gov (NCT06289296 and NCT06368271.

Sample preparation of murine liver, adipose and skeletal muscle tissue. Skeletal muscle from the gastrocnemius, liver, and subcutaneous inguinal white adipose tissue was harvested from C57BL/6NTac (Taconic) mice and flash frozen in liquid nitrogen. The tissues were then pulverized in liquid nitrogen using a mortar and pestle, after which they were stored at – 80 °C. 10 mg of tissue were spiked with a mixture of labeled internal standards (final concentration 10 μM) and 180 μL of 80% aqueous methanol with 0.1 M formic acid were added to the sample. In the following step, the samples were vortex mixed for 5 s and neutralized with 20 μL of 9% ammonium bicarbonate and vortexed for 5 s. A tungsten bead was added into each sample and shaken at 30 kHz for 3 min in a mixer mill MM 400 (Retsch, Haan, Germany). After the protein precipitation at −20 °C for 30 min, the samples were centrifuged, and supernatants were transferred into LC vials and evaporated in miVac Quattro Concentrator (Genevac, UK). Finally, the samples were reconstituted in 50 μL of 50% aqueous methanol. The gastrocnemius muscles for analysis of acyl-CoA were rapidly excised from male control (PanK4flox/flox) mice (15-25 weeks of age, n = 8), as previously described[3]. The extraction of acyl-CoA from murine muscles was modified to increase extraction yield and preserve these unstable metabolites during the extraction process. Briefly, 30 mg of skeletal muscle were spiked with 10 μL of 50 mM ascorbic acid and 10 μL of

50 μM isotopically labeled internal standards (Ac-CoA-$^{13}C_2$, Mal-CoA-$^{13}C_3$), then 360 μL of 0.1 M formic acid in 80% aqueous methanol were added and the samples were vortex mixed for 5 s. Following this, the samples were neutralized by addition of 40 μL of 9% ammonium bicarbonate and vortexed mixed for 5 s. Eventually, the samples were shaken with a tungsten bead at 30 kHz for 3 min and centrifuged at 14,000 *g* for 10 min. The supernatants were evaporated in a vacuum concentrator and redissolved in 50 μL of 50% aqueous methanol. For analysis of the NAD cofactors, 10 μL of supernatant before drying was 10× diluted with 80% acetonitrile.

Sample preparation of bacterial extracts. Bacterial cultivation and sample processing were performed as described earlier[1]. *E. coli* K12 strain was grown in lysogeny broth (LB) media prior to the experiment at 37 °C. To prepare *E. coli* inoculum, a fresh bacterial colony on LB agar media was transferred into 2 L of MOPS medium (40 mM MOPS/4 mM Tricine adjusted to pH 7.4 with KOH, 1 mM glucose, 9.5 mM NH_4Cl, 0.27 mM K_2SO_4, 1.3 mM K_2HPO_4, 0.52 mM $MgCl_2$, 50 mM NaCl, 10 μM $FeSO_4$, 0.002 μM $(NH_4)_6Mo_7O_{24}$, 0.4 μM H_3BO_3, 0.03 μM $CoCl_2$, 0.009 μM $CuSO_4$, 0.08 μM $MnCl_2$, 0.001 μM $ZnSO_4$) and incubated for 18 hr at 37 °C and 220 rpm in Innova 44 incubator shaker (New Brunswick, USA). A 250 mL Erlenmeyer flask containing 50 mL of MOPS medium was inoculated to an initial OD600 of 0.05. The bacterial suspension was cultivated at 37 °C with shaking at 250 rpm until it reached the exponential phase, determined by OD600 ~ 0.5. To trigger the RelA-mediated stringent response, cells were treated with 150 μg/mL mupirocin (pseudomonic acid) for 5 minutes before sample collection. One more timepoint (0 min) was collected before induction of stringent response (addition of mupirocin). Depending on the type of experiment, 10–13 mL of cell suspension was rapidly collected from the cultivation flask and vacuum filtered through a 25 mm, 0.45 μm cellulose acetate filter. The filter membrane, containing the retained bacteria, was immediately transferred to a 1.5 mL microtube containing 0.6 mL of ice-cold 1 M acetic acid and then frozen in liquid nitrogen. Samples exceeding the time of both filtration and freezing more than 15 s were discarded. For each point of analysis three parallel samples were collected to estimate precision of the process extraction. The samples were subsequently thawed slowly on ice and

incubated for 30 minutes with brief vortexing every 5 minutes. The crude bacterial lysate was separated from the cellulose acetate filter by short centrifugation (8000 *g* for 30 seconds) through a pinhole at the bottom of a 1.5 mL microtube, which was assembled with a 2 mL collection tube. The lysate was quickly frozen in liquid nitrogen and lyophilized. Before the LC-MS analysis, samples were reconstituted in 100 µL of 50% aqueous methanol.

Culture of mouse pluripotent stem cells. Mouse embryonic stem cells (mESCs) E14J (Joshua Brickman Lab, reNEW, University of Copenhagen) were maintained in naive conditions on fibronectin-coated (Corning) tissue culture plates in N2B27 media consisting of a 1:1 mixture of DMEM/F12+Glutamax (Gibco, Cat#10565018) and Neurobasal (Gibco, Cat#21103-049), supplemented with 1xN2 (Gibco, Cat#17502048), 1xB27 (Gibco, Cat#17504044), 1 mM Glutamax (Gibco, Cat#35050061), 100 µM b-mercaptoethanol (ThermoFisher Scientific, Cat#M3148) and 2iLIF: 3 µM CT99021 (Axon Medchem, Cat#1386), 1µM PD (Axon Medchem, Cat#1408) and 10 ng/mL LIF (made in house). The cells were cultured under hypoxic conditions (5% O2, 5% CO2) at 37°C and passaged every 2-3 days.

For the [U-13C]glucose labelling metabolomics experiments, ESCs were cultured in regular N2B27 with 2iLIF. Five hours prior to harvesting, the media were replaced with N2B27 consisted of a 1:1 mixture of DMEM/F12 depleted of glucose, glutamine, glutamate and pyruvate (Sartorius/In vitro, Cat#06-1170-94-1A) and Neurobasal without glucose and sodium pyruvate (Gibco, Cat#A2477501), supplemented with [U-13C]glucose (Merck Sigma-Aldrich, Cat#389374) and unlabeled glutamine (Glutamax, Gibco, Cat#35050061) and pyruvate (Gibco, Cat#11360070).

Harvesting and intracellular metabolite extractions: Cells were washed three times with PBS and quenched by adding 0.25 mL ice-cold 90% methanol, followed by collection via scraping. The samples were stored at -80°C until extraction. For intracellular metabolite extraction, samples were thawed on ice, snap-frozen in liquid nitrogen, thawed again and vortexed. This freeze-thaw-vortex cycle was repeated three times. Samples were then incubated on ice for 1 hour and centrifuged at 15,000 rpm for 15 minutes at 4°C. A total of 0.15 mL of the supernatant was collected for downstream analysis. The labelling was

calculated according to previously published methodology with an in-house script, where natural occurrence and unlabelled control sample were taken into an account[4,5].

LC-MS method for lipid analysis. The analysis of lipids was performed on Agilent 1290 UHPLC coupled with Agilent 6546 qTOF mass spectrometer. For the lipidomic separation, ACQUITY UPLC CSH C18 Column, 50 × 2.1 mm, 1.7 µm (Waters, USA) was used, the column and autosampler were kept at 65 °C and 10 °C, respectively. Mobile phase A was 60% aqueous acetonitrile and mobile phase B contained 10 mM ammonium formate in 89.1% 2-propanol, 10.5% acetonitrile, 0.4% water and 0.1% formic acid. The flow rate was 0.5 mL/min with the following gradient: 0 min (15% B), 1.2 min (30% B), 1.5 min (55% B), 5 min (55% B), 7 min (72% B), 9.5 min (85% B), 10 min (100% B), 13.1 min (100% B), 13.4 min (15% B), 14.3 min (15% B). Lipids were ionized in positive mode in an electrospray ion source. The source and gas parameters were set as follows: ion spray voltage +4.0 kV, gas temperature 150 °C, drying gas flow 8 L/min, nebulizer pressure 35 psi, sheath gas temperature 350 °C, sheath gas flow 11 L/min, fragmentor 120 V, mass range 100-1700 *m/z*. The data was evaluated by Profinder 10.0.2 (Agilent, CA, USA).

Method validation for separation of NAD metabolite. The *sensitivity* of the method was assessed and the LOD values for all NAD metabolites were determined as a concentration corresponding to a signal-to-noise ratio (*S*/*N*) of 3 and the LOQ values were determined as a *S*/*N* of 10. *Linearity* of the method was evaluated through a 14-point (n = 3) linear regression model yielding the calibration curves constructed by using internal calibration with isotopically labeled standards prepared in pure solvents. Due to the lack of isotopically labeled standards for $NADP^+$ and NADPH, NAD-D_4 and NADH-D_4 were used for quantification of $NADP^+$ and NADPH, respectively. The *Accuracy* of quantitation was evaluated through analysis of a real extract and the same extract spiked with standard solution at three concentration levels (0.3 µM, 0.5 µM and 1 µM standard addition); eventually, concentration in an original sample was subtracted from the total concentration in a spiked sample and accuracy was calculated as [(mean observed concentration)/(spiked concentration)] × 100%. *Precision* was calculated as RSD of quantitation

for 7 repeated measurements of one extract. The *carry-over* effect was evaluated based on the presence of analytes in the blank solvent, which was injected right after the analysis of the calibration solution with the highest concentration (10 µM). *Repeatability* in peak areas was determined as RSD of repeated measurements (n = 7) of one of the extracts. *Matrix effects* were assessed by spiking of 1-µM standards of NAD metabolites in the extracts (n = 3). The matrix effects were calculated by subtracting the peak areas in the original extracts from the peak areas in the spiked extracts and compared to the peak areas of 1-µM pure standards according to the following equation:

$$ME = \left(\frac{S - O}{Std}\right) \times 100$$

Where *ME* represents matrix effect (%), *S* is peak area in spiked extract, *O* is peak area in an original extract, and *Std* is peak area of a pure standard.

The *stability* of the metabolites in extracts was evaluated by comparing their peak areas measured immediately after extraction versus peak areas measured after 24 hours in the LC autosampler at 6 °C. Stability of individual NAD metabolite in acidic and basic solvents at ambient temperature (21 °C) was also tested. Each NAD metabolite was dissolved in three different solvents - (1) 0.1 M sodium hydroxide, (2) 0.6 M perchloric acid, and (3) 0.1 M formic acid in 80% aqueous methanol – to a final concentration of 1 mM. After certain timepoints (0.3 h, 2 hr, 21 hr, and 45 hr) an aliquot of each solution was neutralized with 9% ammonium bicarbonate solution and 100× diluted with 80% aqueous acetonitrile before injection to LC-MS/MS. The NAD metabolites and their degradation products including AMP, ADP, ATP, NAM, NMN, and ADPR were monitored in the MRM mode.

Method validation for separation of nucleotides and their derivates. The validation was performed on samples of human plasma extracts following the ICH guideline M10 on bioanalytical method validation and study sample analysis[6]. The validation was evaluated based on several parameters, including the limit of quantification (LOQ), linearity, accuracy, precision, matrix effects, repeatability, stability, carry-over, and recovery. Recovery was determined on human plasma samples based on comparison of spiked

samples before extraction vs after extraction and calculated as: (concentration before/concentration after × 100). The presence of carry-over effect was evaluated based on the presence of analytes in the blank solvent, which was injected right after the analysis of the calibration solution with high concentration (10 μM). Matrix effects were tested by spiking nucleotide standards at two concentration levels (1 μM and 10 μM) into the samples of human plasma obtained from 5 individual donors. For the evaluation of matrix effect, peak areas of spiked samples were compared with peak areas of the pure mix of standards and calculated by using equation:

$$ME = (\frac{S - O}{Std}) \times 100$$

ME - the matrix effect (%), *S* is peak area in spiked extract, *O* is peak area in an original extract, and *Std* is peak area of a pure standard.

Precision was evaluated based on the quantification RSD values for 4 consecutive measurements of human plasma sample. Accuracy was calculated based on the measurement of human plasma and the same sample spiked with standards at two concentration levels (5 μM and 10 μM) and calculated by using equation:

$$Accuracy = \left(\frac{Co}{Cs}\right) \times 100\ \%$$

Co - observed concentration, *Cs* - concentration of standard spiked into the sample.

The LOQ values were determined as a concentration at which the analyte was quantified with an accuracy of 100 ± 20%. The recovery and stability of acyl-CoAs were tested on 10 μM mixture of standards that were extracted with 360 μL of 0.1 M formic acid in 80% methanol with and without addition of 10 μL of 50 mM ascorbic acid by (i) manual glass homogenizer and (ii) shaking with a bead in a bead mill at 30 kHz for 3 min. Samples were neutralized with addition of 40 μL of 9% ammonium bicarbonate after short homogenization with the glass homogenizer and then homogenized further. In the plastic tubes, the

samples were neutralized after approximately 5 seconds of vortexing. Eventually, the samples were dried in glass LC-microvials and redissolved in 50 µL of 1 mM ascorbic acid and 5 mM ammonium acetate in 50% methanol.

The linearity was evaluated through 14-point internal calibration with isotopically labelled standards for most compounds, external calibration was used for the metabolites whose analogues were not available. All calibrations were prepared in pure solvents and 1/x weighting factor was applied to all calibration curves.

Recovery of nucleotides from human plasma (n = 5) was determined through analysis of human plasma spiked with 10 µM standards before extraction and compared to concentrations calculated from human plasma spiked with 10 µM standards after extraction according to the following formula:

$$Recovery = \left(\frac{C1}{C2}\right) \times 100\ \%$$

$C1$ – concentration in pre-extraction spiked sample, $C2$ - concentration in post-extraction spiked sample.

Stability testing of standard nucleotides. The stability was tested on a mixture of ATP, GTP, UTP and CTP standards diluted into three different solvents – water, 50% aqueous acetonitrile and 50% aqueous methanol (c = 100 µM). Aliquots of freshly prepared samples were measured by LC-UV-MS/MS and the stock solution was stored at -20 °C. Every week, these stock solutions were thawed, and their aliquots were 10× diluted with 50% aqueous methanol and analyzed. Stability of triphosphates was evaluated based on the monitoring of percentage ratio between triphosphates and emerging di- and monophosphates over the course of 14 weeks.

Table S2 Description of the scoring system for calculation of the quality score

$$Quality\ Score = \sum_{i=1}^{n} Peak\ shape\ score + \sum_{i=1}^{n} Peak\ intensity\ score$$

Peak shape score	Peak intensity score
0 – No peak<br>1 – tailing factor > 2 or < 0.8<br>2 – tailing factor < 2 or > 0.8	0 – $S/N < 3$<br>1 – S/N between 3 and 10<br>2 – $S/N > 50$

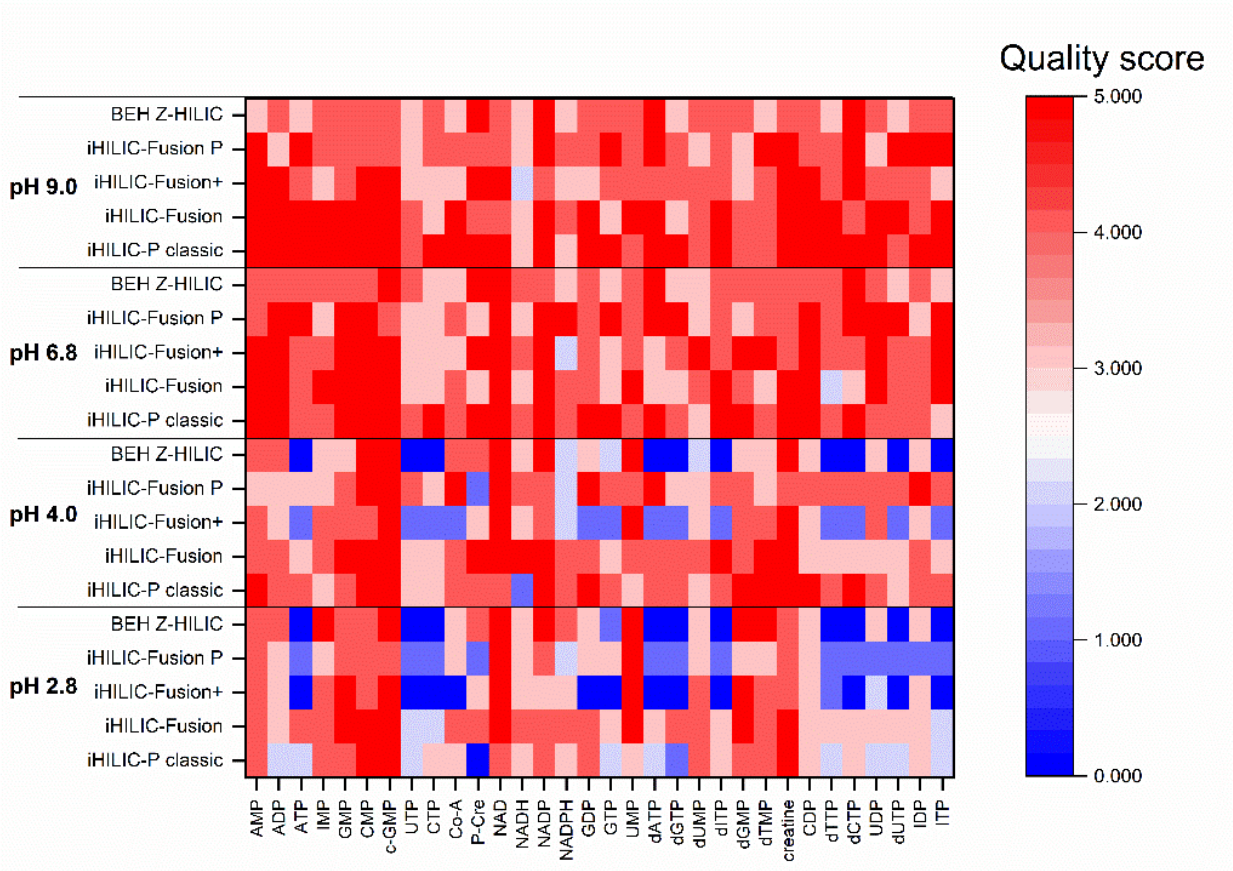

Figure S1 Quality score calculated from signal-to-noise ratio and tailing factor of a mixture of standards analyzed on 5 tested columns under various pH of buffer in the mobile phase.

Table S3 LC-MS parameters for NAD metabolites with respective validation parameters such as limits of detection [LOD], limits of quantification [LOQ], linear calibration range with coefficient of determination (R^2), precision, repeatability in peak areas, and matrix effects.

Metabolite	t_R [min]	Precursor	Product	Collision energy [V]	LOD [nM]	LOQ [nM]	Calibration range [nM] (R^2)	Precision [%]	Matrix effects [%]	Accuracy [%] Low 0.3 µM	Medium 0.5 µM	High 1 µM
NADH	0.69	666	108	77	0.5	1	1-10000 (0.9950)	4.8	3.4	108.6	109.1	110.2
NAD	0.93	664	136	53	0.5	1	1-10000 (0.9954)	2.5	−6.0	105.1	97.7	110.8
NADPH	1.55	746	136	57	10	25	25-10000 (0.9903)	3.9	7.4	100.6	106.4	97.5
NADP	1.87	744	136	66	3	10	10-10000 (0.9940)	4.5	2.6	103.0	99.6	100.2
NADH-D4	0.69	670	112	77								

NAD-D4	0.93	668	136	53		
ADPR	0.804	560	136	42	1	5
NMN	1.97	335	123	13	30	80
NAM	0.24	123	80	25	30	60
AMP	0.98	348	136	17	3	15
ADP	1.32	428	136	29	15	40
ATP	1.54	508	136	39	20	60

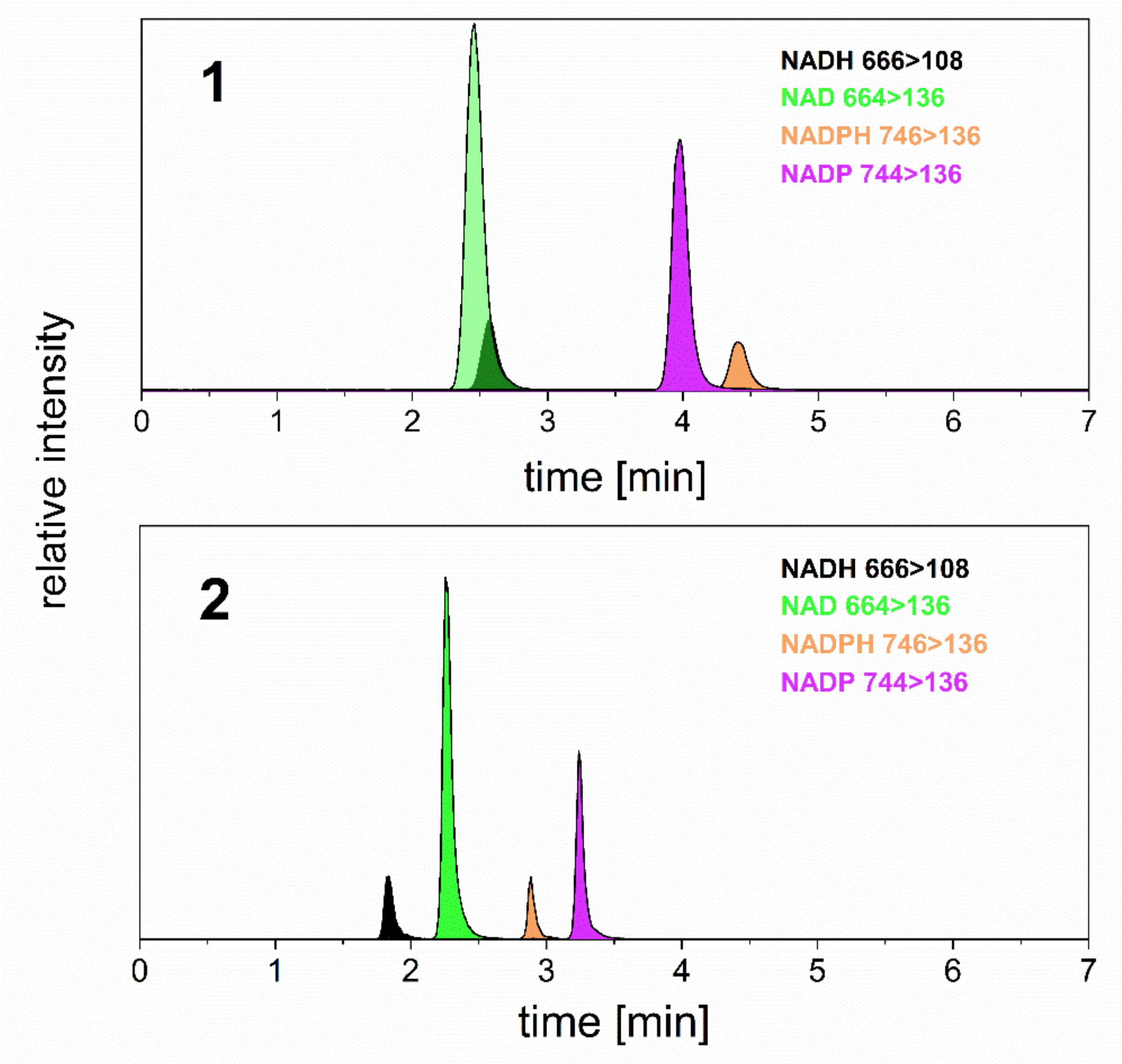

Figure S2 Comparison of separation on (**1**) iHILIC-(P) Classic, PEEK, 50×2.1, 5 µm and (**2**) iHILIC-Fusion, SS, 50×2.1, 1.8 µm. Mobile phase: (A) 10 mM ammonium acetate with 5 µM medronic acid in water, (B) 10 mM ammonium acetate in 90 % aqueous acetonitrile. Gradient elution: 0 min (85% B), 5 min (60% B), 7 min (30% B), 8 min (30% B), 9 min (85% B), 15 min (85% B).

Table S4 LC-MS/MS parameters for nucleotides and their derivates.

Metabolite	t_R [min] (position) iHILIC - (P) Classic 30 x 2.1 mm	t_R [min] (position) iHILIC - (P) Classic 50 x 2.1 mm	Precursor	Product	Collision energy [V]	Polarity

3-dp-CoA	1.14 (3)	1.38 (1)	688	261<br>348	25<br>21	Positive
dTMP	1.33 (4)	1.54 (2)	323	81<br>53	25<br>69	Positive
cyclocreatine	0.94 (1)	1.55 (3)	144	98<br>56	17<br>33	Positive
creatine	1.06 (2)	1.74 (4)	132	90<br>87	16<br>16	Positive
c-GMP	1.43 (5)	2.04 (5)	346	152<br>110	29<br>57	Positive
dUMP	1.55 (6)	2.06 (6)	309	81<br>53	5<br>61	Positive
c-di-AMP	1.66 (8)	2.28 (7)	659	524<br>330	25<br>25	Positive
AMP	1.64 (7)	2.34 (8)	348	136<br>97	17<br>41	Positive
Ac-CoA	1.89 (10)	2.43 (9)	810	303<br>428	33<br>25	Positive
UMP	1.87 (9)	2.70 (10)	325	97<br>41	9<br>53	Positive
AICAR	1.98 (11)	2.80 (11)	339	110<br>127	45<br>12	Positive
IMP	2.02 (12)	2.84 (12)	349	137<br>110	13<br>57	Positive
dGMP	2.09 (13)	2.90 (13)	348	152<br>135	13<br>49	Positive
CoA	2.27 (15)	2.91 (14)	768	261<br>428	33<br>25	Positive
CMP	2.25 (14)	3.17 (15)	324	112<br>95	17<br>61	Positive
P-creatine	2.43 (16)	3.29 (16)	212	114<br>90	13<br>17	Positive
ADP	2.49 (18)	3.31 (17)	428	136<br>348	29<br>17	Positive
GMP	2.46 (17)	3.41 (18)	364	152<br>135	17<br>49	Positive
dCDP	2.82 (19)	3.73 (19)	388	112<br>95	22<br>80	Positive
dATP	2.87 (22)	3.75 (20)	492	136<br>81	45<br>45	Positive
UDP-Glc	2.82 (20)	3.76 (21)	565	323<br>79	25<br>77	Negative
UDP	2.84 (21)	3.86 (22)	403	79<br>159	65<br>33	Negative
dTTP	2.96 (23)	3.87 (23)	483	81<br>53	33<br>80	Positive
IDP	2.97 (24)	3.89 (24)	429	137<br>97	22<br>34	Positive
CDP	3.17 (25)	4.16 (25)	404	112<br>95	21<br>80	Positive
ATP	3.2 (26)	4.21 (26)	508	136<br>410	39<br>17	Positive
Suc-CoA	3.42 (30)	4.26 (27)	868	361<br>428	41<br>29	Positive
c-di-GMP	3.31 (28)	4.27 (28)	691	152<br>540	42<br>18	Positive
dUTP	3.25 (27)	4.28 (29)	467	159<br>79	41<br>77	Negative
dITP	3.36 (29)	4.44 (30)	493	217<br>81	9<br>45	Positive
GDP	3.46 (31)	4.53 (31)	444	152<br>135	17<br>57	Positive
Mal-CoA	3.76 (36)	4.58 (32)	854	347<br>303	34<br>42	Positive

8-oxo-dATP	3.52 (32)	4.59 (33)	508	152<br>81	25<br>33	Positive
dCTP	3.62 (33)	4.62 (34)	468	112<br>192	30<br>9	Positive
UTP	3.69 (34)	4.79 (35)	483	159<br>79	33<br>80	Negative
ITP	3.75 (35)	4.83 (36)	509	137<br>97	22<br>42	Positive
dGTP	3.86 (37)	4.95 (37)	508	152<br>135	17<br>80	Positive
8-oxo-dGTP	4.0 (38)	5.16 (38)	524	81<br>168	25<br>37	Positive
CTP	4.02 (39)	5.25 (39)	484	112<br>95	33<br>80	Positive
GTP	4.30 (40)	5.62 (40)	524	152<br>135	41<br>80	Positive
ppGpp	4.35 (41)	6.55 (41)	602	504<br>424	20<br>30	Negative
pppGpp	4.46 (42)	6.81 (42)	682	584<br>504	20<br>30	Negative
ADP $^{15}N_5$	2.47	3.31	433	141	29	Positive
Ac-CoA $^{13}C_2$	1.89	2.43	812	305	38	Positive
ATP $^{13}C_{10}$ $^{15}N5$	3.21	4.21	523	146	39	Positive
AMP $^{13}C_{10}$ $^{15}N_5$	1.65	2.34	363	146	17	Positive
CDP $^{13}C_9$ $^{15}N_3$	3.18	4.16	416	119	21	Positive
CMP $^{13}C_9$	2.25	3.17	333	116	17	Positive
Creatine D_3	1.06	1.74	135	93	17	Positive
CTP $^{15}N_3$	4.03	5.25	487	115	33	Positive
dATP $^{13}C_{10}$ $^{15}N_5$	2.87	3.75	507	146	45	Positive
dCDP $^{15}N_3$	2.82	3.73	391	115	22	Positive
dCTP $^{13}C_9$ $^{15}N3$	3.64	4.76	480	119	30	Positive
dGMP $^{13}C_{10}$ $^{15}N5$	2.09	2.90	363	162	13	Positive
dGTP $^{13}C_{10}$ $^{15}N5$	3.85	5.05	523	162	17	Positive
dTTP $^{13}C_{10}$	2.97	4.01	493	86	26	Positive
GDP $^{13}C_{10}$ $^{15}N_5$	3.46	4.53	459	162	17	Positive
GMP $^{15}N_5$	2.46	3.41	369	157	17	Positive
GTP $^{13}C_{10}$	4.31	5.62	534	157	41	Positive
IMP $^{15}N_4$	2.03	2.84	353	141	13	Positive
ITP $^{13}C_{10}$ $^{15}N_4$	3.76	4.93	523	146	22	Positive
Mal-CoA $^{13}C_3$	3.74	4.58	857	350	34	Positive
UDP $^{13}C_9$ $^{15}N_2$	2.84	3.86	414	79	65	Negative
UDP-Glc $^{13}C_6$	2.84	3.76	571	323	21	Negative
UMP $^{13}C_9$	1.89	2.70	334	102	14	Positive
UTP $^{13}C_9$ $^{15}N_2$	3.70	4.91	494	159	33	Negative

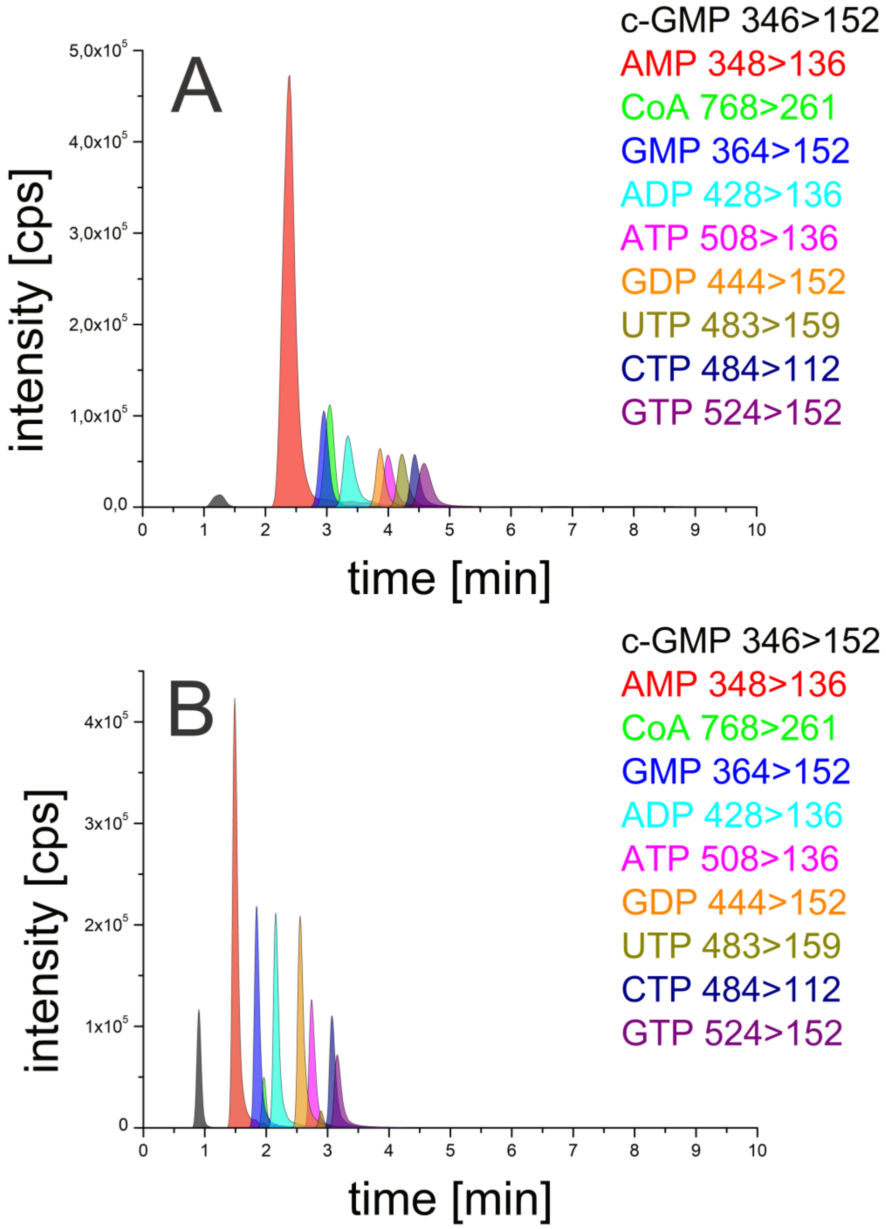

Figure S3 A - Extracted ion chromatograms of 10 µM standard mixture of c-GMP, AMP, CoA, ADP, GMP, ATP, GDP, UTP, CTP and GTP conducted on (A) ACQUITY UPLC BEH Amide, 50×2.1 mm, 1.7 µm, 130Å and (B) ACQUITY UPLC BEH Amide, 30×2.1 mm, 1.7µm, 130Å.

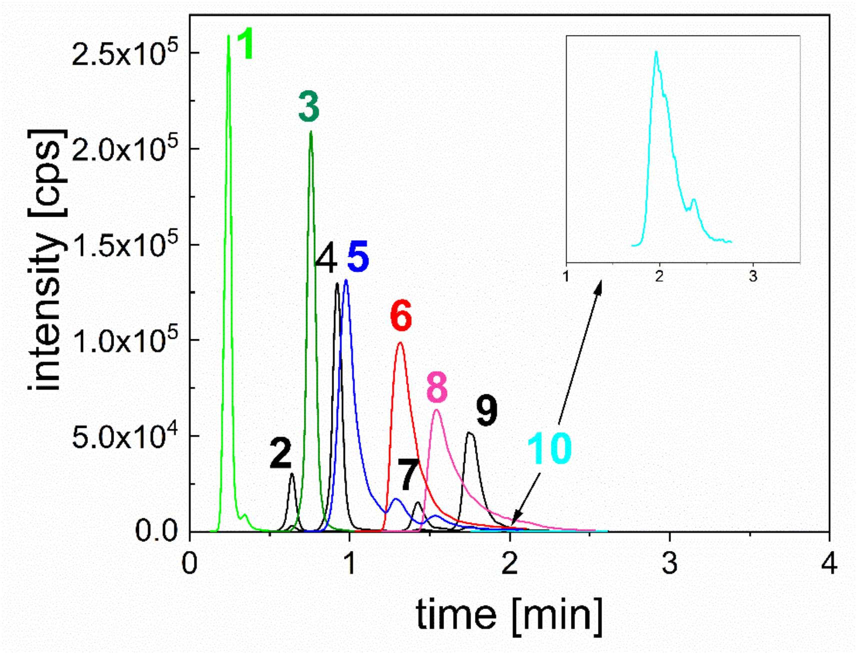

Figure S4 Extracted ion chromatogram of 10 μM standard mixture of NAD metabolites and their degradation products. Peak assignment: 1 – NAM, 2 – NADH, 3 – ADPR, 4 – NAD, 5 – AMP, 6 – ADP, 7 – NADPH, 8 – ATP, 9 – NADPH, 10 – NMN.

Table S5 Degradation of individual NAD metabolites in acidic, alkaline, and neutral conditions with respective degradation products. The percentage in the parentheses corresponds to peak area of individual compound normalized to the sum of all peak areas.

	**Products of degradation**			
	**0.6 M PCA**	**0.1 M FA**	**Neutral**	**0.1 M NaOH**
**NAD^+**	AMP (3 %)<br>ADPR (2.4 %)<br>NAM (2 %)	AMP (0.5 %)<br>ADPR (3 %)<br>NAM (3 %)	AMP (3 %)<br>ADPR (4 %)<br>NAM (0.5 %)	AMP (68 %)<br>ADP (6 %)<br>NAM (25 %)<br>ADPR (1 %)
**NADH**	NAM (0.5 %)<br>ADPR (75 %)	NAM (6.6 %)<br>ADPR (81 %)	ADPR (2.2 %)<br>AMP (0.3 %)	AMP (57 %)<br>NAM (28 %)<br>ADPR (6.8 %)
**NADP**	NAM (6 %)<br>ADP-2'-5' (2 %)<br>ADPR (2 %)	NAM (6 %)<br>ADP-2'-5' (1 %)<br>ADPR (2 %)	NAM (7 %)<br>ADP-2'-5' (0.6 %)<br>ADPR (2 %)	ADP-2'-5' (61 %)<br>NAM (34 %)<br>AMP (2.4 %)<br>ATP-2'-5' (2 %)
**NADPH**	ADP-2'-5' (77 %)<br>NADP (13 %)<br>NAM (4 %)	NAM (63 %)<br>NADP (16%)<br>ADP-2'-5' (7 %)	NADP (15%)<br>NAM (1.4 %)	ADP-2'-5' (59 %)<br>NAM (33 %)

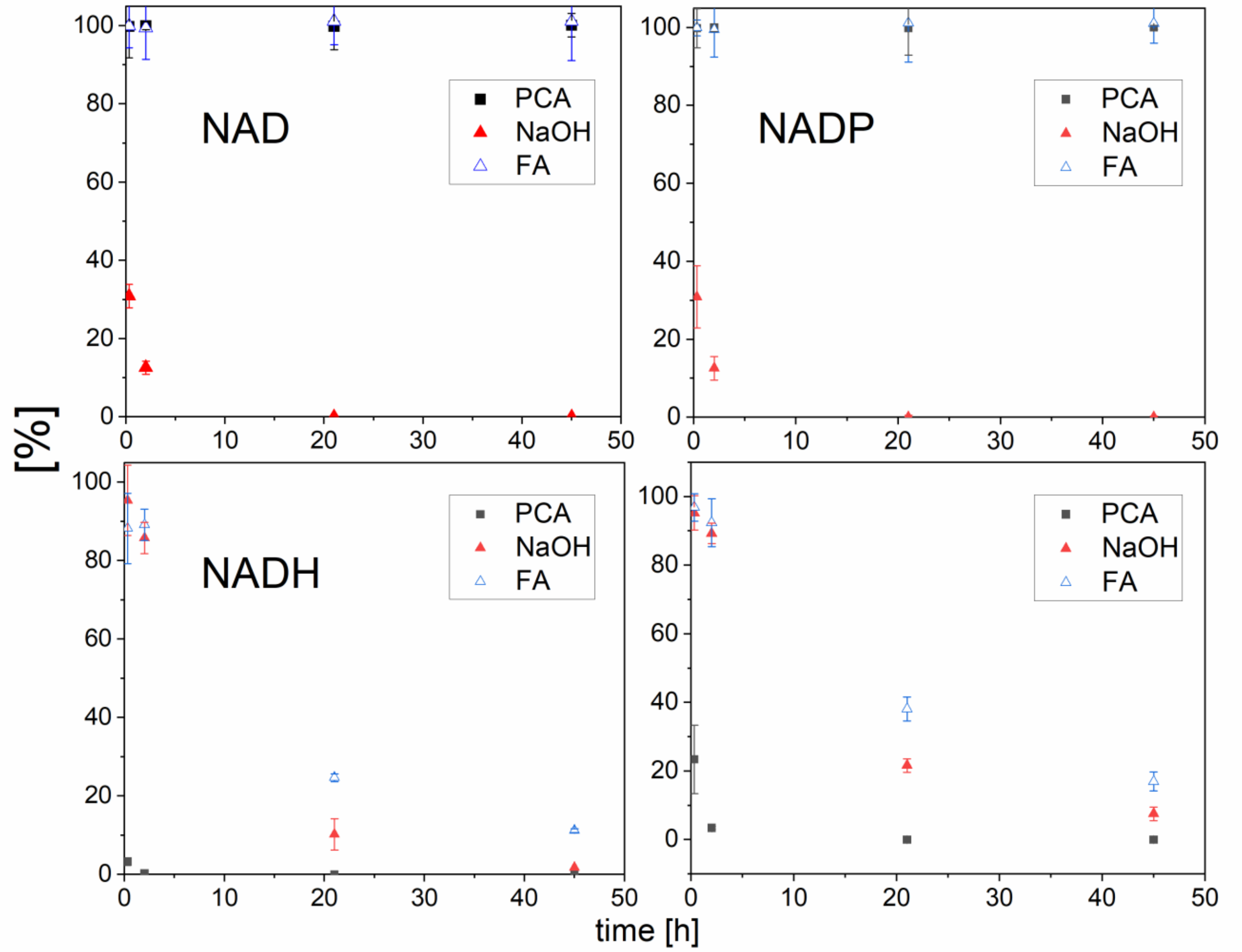

Figure S5 Stability of NAD metabolite standards dissolved in 0.6 M perchloric acid (PCA), 0.1 M sodium hydroxide (NaOH), and 0.1 M formic acid in 80% methanol (FA), average ± SD (*n* = 3). The % degradation corresponds with decrease in peak areas compared to the peak areas obtained from a parallel experiment, where the NAD standards were kept in a neutralized solution (0.1 M formic acid in 80 % methanol neutralized with 9 % ammonium bicarbonate).

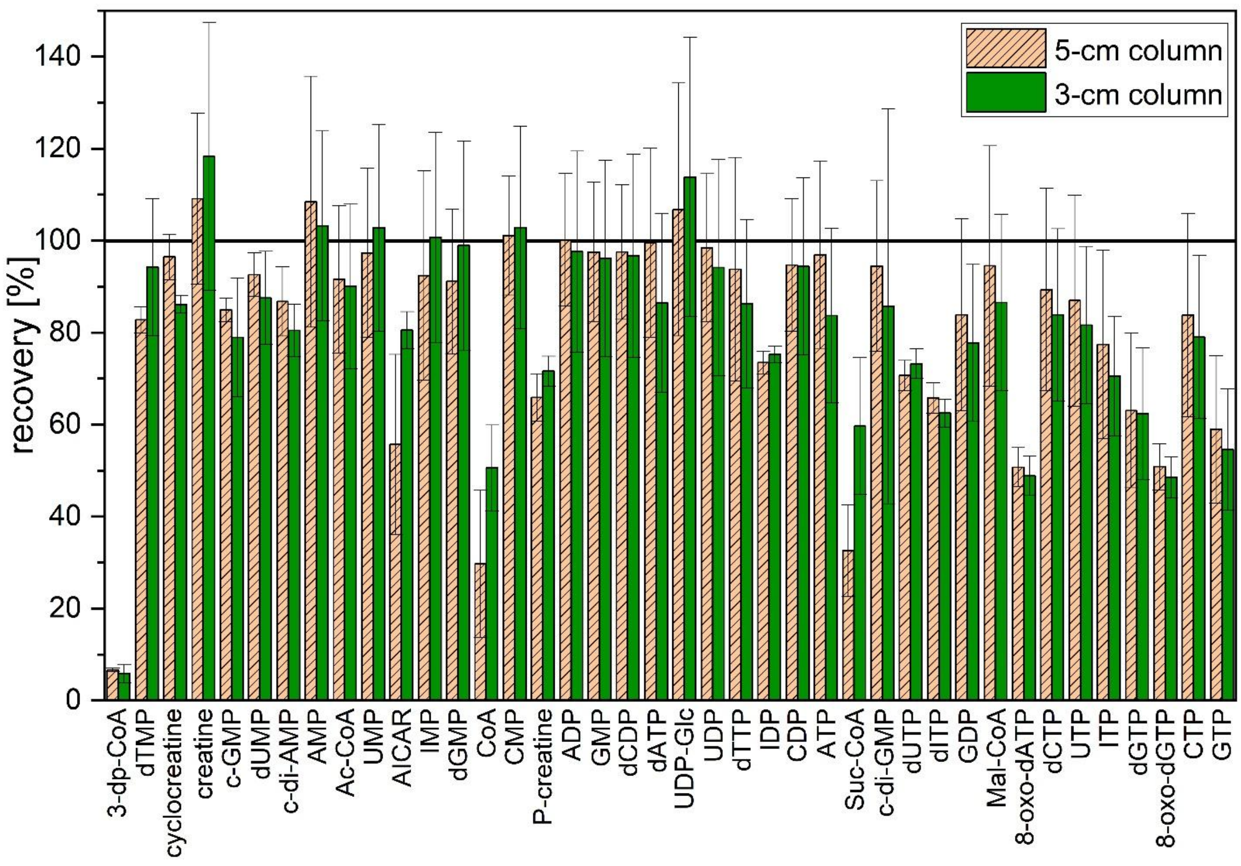

Figure S6 Recovery determined on spiked human plasma and measured by using iHILIC - (P) Classic 50 × 2.1 mm, 5 µm and iHILIC - (P) Classic 30 × 2.1 mm, 5 µm

Table S6 Validation parameters for nucleotides analyzed on iHILIC-(P) Classic, PEEK, 50×2.1, 5 µm with respective limits of quantification [LOQ], linear calibration range, precision, accuracy, and repeatability of peak areas.

Metabolite	LOQ [nM]	Calibration range [µM]	Precision [%]	Accuracy [%]		Repeatability [%]
				5 µM	10 µM	
3-dp-CoA	30	0.03-5	12.7	74.3	93.6	12.7
8-oxo-dATP	30	0.03-50	1.2	155.0	105.1	5.6
8-oxo-dGTP	30	0.03-50	2.9	162.2	125.0	3.6
Ac-CoA	5	0.005-50	2.0	39.5	79.2	6.9
ADP	5	0.005-50	2.5	106.0	106.6	8.4
AICAR	30	0.03-30	7.2	1.2	1.1	6.5
AMP	5	0.005-50	4.0	107.2	106.1	10.0
ATP	5	0.005-50	3.1	107.1	104.2	33.9
c-di-AMP	5	0.005-50	7.4	87.1	90.8	8.0

c-di-GMP	30	0.03-50	1.8	14.5	20.0	0.9
CDP	10	0.01-50	3.2	108.2	108.8	8.6
c-GMP	5	0.05-50	7.6	70.5	60.0	7.6
CMP	5	0.005-50	2.6	105.4	105.9	49.9
CoA	5	0.005-50	-	-	-	-
creatine	5	0.005-50	1.6	108.9	107.6	1.6
CTP	3	0.03-50	2.1	106.4	108.1	6.4
cyclocreatine	5	0.005-10	1.5	345.2	246.1	1.5
dATP	5	0.005-50	3.8	110.9	103.1	13.4
dCDP	5	0.005-50	3.3	104.1	108.4	13.8
dCTP	10	0.01-50	1.9	105.7	104.9	5.2
dGMP	5	0.005-50	7.8	106.6	104.8	13.0
dGTP	30	0.03-50	1.7	106.7	108.0	7.9
dITP	30	0.03-50	12.6	103.2	106.8	3.4
dTMP	30	0.03-10	9.4	90.7	87.0	7.4
dTTP	30	0.03-50	7.1	108.0	104.9	44.6
dUMP	30	0.03-10	7.1	159.0	171.3	4.1
dUTP	30	0.03-50	12.1	8.8	7.9	12.1
GDP	10	0.01-50	5.4	100.3	102.0	6.8
GMP	5	0.005-50	2.0	110.7	105.1	8.4
GTP	30	0.03-50	3.2	98.3	107.0	4.8
IDP	50	0.05-50	20.5	39.5	35.7	15.8
IMP	10	0.01-50	4.2	106.1	108.7	7.2
ITP	70	0.07-50	10.1	106.2	100.7	4.2
Mal-CoA	10	0.5-50	3.0	93.2	105.5	3.5
P-creatine	5	0.005-10	10.3	15.0	13.2	9.6
Suc-CoA	10	-	-	-	-	-
UDP	10	0.01-50	3.3	108.4	105.6	29.3
UDP-Glc	5	0.005-50	6.5	101.4	101.5	21.8
UMP	5	0.005-50	3.5	107.8	112.0	8.4
UTP	30	0.03-50	2.3	101.6	106.9	3.1

Table S7 Validation parameters for nucleotides analyzed on iHILIC - (P) Classic 30 x 2.1 mm, 5 µm with respective limits of quantification [LOQ], linear calibration range, precision, accuracy, and repeatability of peak areas.

Metabolite	LOQ [nM]	Calibration range [µM]	Precision [%]	Accuracy [%]		Repeatability [%]
				5 µM	10 µM	
3-dp-CoA	30	0.03-5	-	-	-	-
8-oxo-dATP	30	0.03-50	7.3	145.7	162.5	7.3
8-oxo-dGTP	30	0.03-50	2.4	111.2	112.3	2.6
Ac-CoA	10	0.01-10	4.0	60.4	101.5	1.5
ADP	5	0.005-50	1.8	97.1	95.7	1.7
AICAR	30	0.03-30	2.7	58.1	71.4	1.6
AMP	5	0.005-50	1.4	110.3	107.4	2.4
ATP	5	0.005-50	2.2	104.6	107.9	3.6
c-di-AMP	5	0.005-50	5.5	81.4	108.9	0.8
c-di-GMP	30	0.03-50	10.8	22.1	31.1	5.6
CDP	10	0.01-50	3.1	110.6	108.5	2.2

c-GMP	10	0.01-50	8.4	85.6	85.0	9.5
CMP	5	0.005-50	3.2	102.6	105.8	4.6
CoA	5	0.005-50	-	-	-	-
creatine	5	0.005-50	1.3	110.7	111.9	9.5
CTP	30	0.03-50	2.4	106.6	112.3	2.6
cyclocreatine	5	0.005-10	1.8	141.3	114.8	1.8
dATP	5	0.005-50	3.3	101.1	106.9	3.2
dCDP	5	0.005-50	6.1	111.1	109.9	2.6
dCTP	10	0.01-50	3.5	92.2	110.7	2.2
dGMP	5	0.005-50	2.6	107.0	107.2	1.7
dGTP	30	0.03-50	1.0	102.6	105.9	3.3
dITP	30	0.03-50	6.3	128.5	184.0	2.8
dTMP	30	0.03-5	16.4	-	-	16.3
dTTP	30	0.03-50	5.9	102.9	104.9	6.0
dUMP	30	0.03-30	1.5	115.0	117.2	2.7
dUTP	30	0.03-50	5.6	37.8	31.5	5.6
GDP	10	0.01-50	2.2	104.2	101.3	2.6
GMP	5	0.005-50	3.8	102.8	110.2	1.1
GTP	30	0.03-50	3.1	106.5	99.0	2.2
IDP	50	0.05-50	8.8	102.6	104.8	7.7
IMP	10	0.01-50	3.6	103.9	107.6	2.7
ITP	70	0.07-50	3.6	96.0	106.0	5.4
Mal-CoA	10	0.01-100	5.3	87.5	107.0	6.7
P-creatine	5	0.005-5	2.9	29.1	20.4	2.9
Suc-CoA	10	0.01-100	-	-	-	-
UDP	10	0.01-50	4.8	110.0	103.5	3.2
UDP-Glc	5	0.005-50	4.4	107.6	106.2	4.5
UMP	5	0.005-50	1.6	112.6	111.5	1.3
UTP	30	0.03-50	4.5	97.3	101.1	1.6

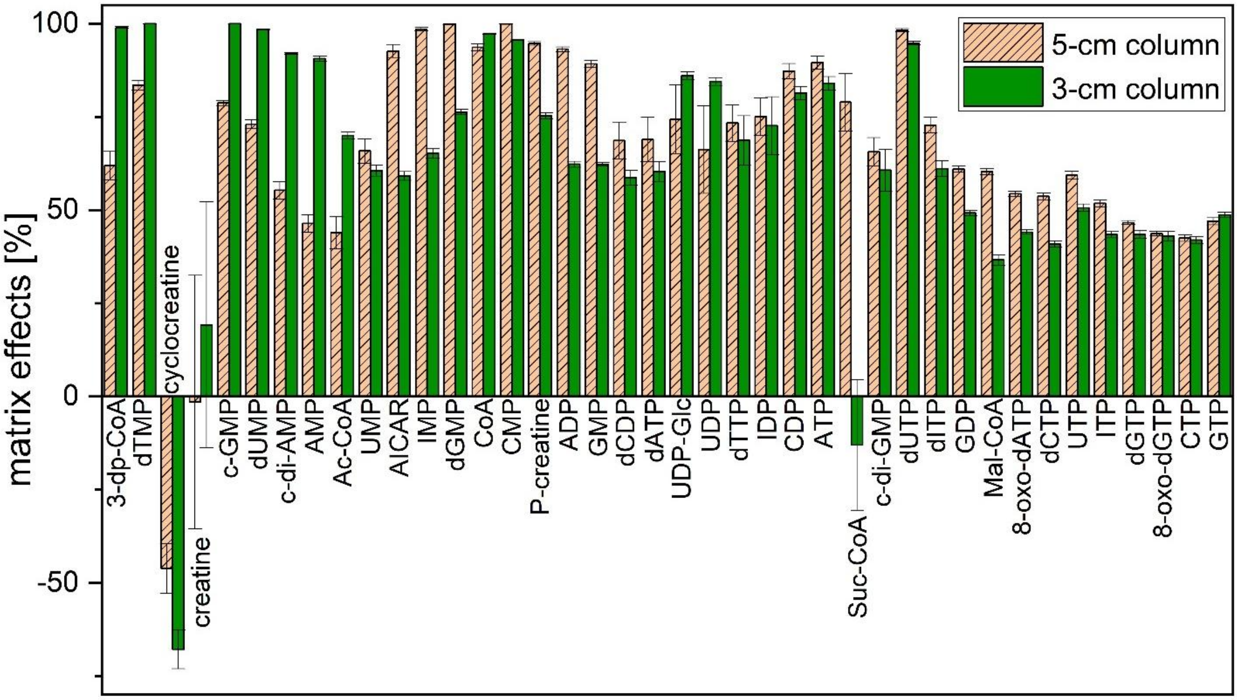

Figure S7 Comparison of matrix effects in spiked human plasma measured on iHILIC - (P) Classic 50 × 2.1 mm, 5 µm and iHILIC - (P) Classic 30 x 2.1 mm, 5 µm.

Table S8 Concentration in 10 mg of murine liver (*n* = 10), murine skeletal muscle (*n* = 5), and murine white adipose tissue (*n* = 5), median ± confidence interval at a confidence level α = 0.95 with RSD in % in parentheses

	**Murine liver by LC-MS/MS [pmol/mg]**	**Murine liver by cycling assay [pmol/mg]**	**Reference values in murine liver from literature[43] [pmol/mg]**	**Murine skeletal muscle by LC-MS/MS [pmol/mg]**	**Murine adipose tissue by LC-MS/MS [pmol/mg]**
NADH	134 ± 27 (23)	168 ± 18 (18)	1 - 109	144.2 ± 28.8 (25.9)	3.0 ± 0.4 (16.1)
NAD	943 ± 123 (15)	1309 ± 64 (8)	2 - 1132	698.3 ± 80.2 (12.5)	35.1 ± 5.8 (20.1)
NADPH	63 ± 13 (24)	190 ± 17 (14)	28 – 237	13.8 ± 2.6 (20.5)	8.0 ± 1.7 (25.7)
NADP	75 ± 14 (21)	89 ± 6 (12)	50 – 247	29.8 ± 5.7 (22.3)	3.9 ± 0.9 (26.7)

Table S9 Concentrations of nucleotides and other phosphorylated metabolites in murine tissues, human plasma and E. coli quantified on 3-cm and 5-cm iHILIC-(P) Classic, PEEK, 50×2.1, 5 µm. Concentration displayed as mean concentration ± confidence interval at confidence level 0.95

	Murine muscle [pmol/mg]		Murine liver [poml/mg]		Murine white adipose tissue [pmol/mg]		Human plasma [nM]		E. coli untreated with mupirocin [µM]	
**metabolite**	**3-cm**	**5-cm**	**3-cm**	**5-cm**	**3-cm**	**5-cm**	**3-cm**	**5-cm**	**3-cm**	**5-cm**
3-dp-CoA	<LOQ	<LOQ	0.86±0.15	1.8±0.5	<LOQ	0.2±0	<LOQ	<LOQ	<LOQ	<LOQ
dTMP	<LOQ	<LOQ	<LOQ	<LOQ	<LOQ	<LOQ	<LOQ	<LOQ	<LOQ	136.7±52.8
cyclocreatine	<LOQ	<LOQ	0.05±0.01	0.04±0.01	0.003±0.0009	<LOQ	<LOQ	<LOQ	<LOQ	<LOQ
creatine	36296.2±4058.4	34703.3±3713.6	242.4±36	233.9±36.6	285±42.8	275.5±40	25.9±0.4	26.4±0.6	2.5±0.5	2.7±0.2
c-GMP	<LOQ	<LOQ	<LOQ	<LOQ	<LOQ	<LOQ	<LOQ	<LOQ	<LOQ	<LOQ
dUMP	<LOQ	<LOQ	<LOQ	1.4±0.1	<LOQ	<LOQ	<LOQ	0.11±0.01	<LOQ	114±8.4
c-di-AMP	<LOQ	<LOQ	<LOQ	<LOQ	<LOQ	<LOQ	<LOQ	<LOQ	<LOQ	<LOQ
AMP	456.7±150	456.9±144.6	1655.9±256.8	1648.4±230.4	131.5±27.2	125.9±25.1	5.5±0.1	5.5±0	1042.3±102	1010.9±100.1
acetyl-CoA	1.8±0.4	1.8±0.4	29.9±3.8	28.5±4.2	0.57±0.06	0.57±0.06	<LOQ	<LOQ	1822.7±49.5	1756.3±27.1
UMP	10.2±1.8	10.5±2.1	502.8±88.4	496.9±91.9	21±3.3	20.9±3.3	<LOQ	<LOQ	402.6±51.4	405.2±35.2
AICAR	0.24±0.01	0.29±0.01	1.4±0.1	1.9±0.1	0.23±0.01	0.19±0.04	0.03±0	0.07±0	715.2±54.5	988.2±89.5
IMP	2279±299.9	2242.1±295.9	665.4±127.8	643.8±112.6	18.8±2.9	18.2±2.9	0.41±0.02	0.38±0.03	307.4±17.4	304.6±15.2
dGMP	0.03±0.02	<LOQ	0.10±0.02	<LOQ	0.004±0.002	<LOQ	<LOQ	<LOQ	4.6±0.6	5.5±0.4
CoA	<LOQ	<LOQ	<LOQ	<LOQ	<LOQ	<LOQ	<LOQ	<LOQ	<LOQ	<LOQ
CMP	3.35±0.49	<LOQ	16.7±3.6	9.2±1.2	1.3±0.3	0.96±0.25	<LOQ	<LOQ	302.7±4.3	215.1±42.9
P-creatine	1017.2±182.2	569.9±128.7	3.4±1.8	4.7±1.3	14.3±1.1	11.4±1	0.5±0.1	<LOQ	<LOQ	<LOQ
ADP	5615.5±723.5	4710.3±1805.5	2299.1±615.3	2295.6±648.1	75.8±7.7	76.1±9.1	2.2±0	1.8±0.2	2278.9±142.3	1821.1±129.6
GMP	8.1±1.3	11.3±6.2	343.9±70.2	337.1±69.3	18.5±3.3	18.3±3.2	0.12±0.01	0.11±0.01	342.9±36.6	354.6±37.8
dCDP	0.07±0.02	0.16±0.03	0.12±0.02	0.15±0.03	<LOQ	0.07±0.01	<LOQ	<LOQ	55.7±4.4	50.8±3.5
dATP	0.49±0.05	0.56±0.08	<LOQ	<LOQ	<LOQ	<LOQ	<LOQ	<LOQ	911.5±39.7	909.3±46.5
UDP-Glc	8.4±1.2	8.8±1.4	170.6±30.3	178.7±30.8	12.4±1.1	12.5±1.3	0.03±0.003	0.04±0.001	8104.5±292.6	8471.9±169.1
UDP	51.5±10.8	57.7±13.2	57.9±16.4	73.5±19.8	9.2±1	10.4±0.9	<LOQ	<LOQ	1029.9±182.9	1308.1±178
dTTP	1.1±0.3	1.2±0.2	<LOQ	<LOQ	<LOQ	<LOQ	<LOQ	<LOQ	566.3±23.4	560.3±15.7
IDP	0.36±0.19	1±0.3	0.12±0	0.54±0.1	0.07±0.02	0.2±0.02	<LOQ	<LOQ	<LOQ	<LOQ
CDP	38.1±10.3	39.2±9.4	6.7±2	7.1±2.1	1.5±0.4	1.6±0.4	<LOQ	<LOQ	240.7±21.8	238.2±19.6
ATP	3816±705.6	3387.5±333.9	695.4±202.2	649.7±189.1	37±10.6	35.3±10	1.9±0.1	1.6±0.1	11839.3±323.7	12010.3±410.5
c-di-GMP	<LOQ	<LOQ	<LOQ	<LOQ	<LOQ	<LOQ	<LOQ	<LOQ	<LOQ	<LOQ
dUTP	<LOQ	<LOQ	<LOQ	<LOQ	<LOQ	<LOQ	<LOQ	<LOQ	<LOQ	<LOQ
dITP	<LOQ	<LOQ	<LOQ	<LOQ	<LOQ	<LOQ	<LOQ	<LOQ	<LOQ	<LOQ
GDP	281±45.6	321.6±86.7	222.7±55.7	241.9±57.3	26.6±3.4	27.7±3.6	0.5±0.01	0.5±0.01	1904.9±160.1	1910.1±138.5
8-oxo-dATP	<LOQ	<LOQ	<LOQ	<LOQ	<LOQ	<LOQ	<LOQ	<LOQ	<LOQ	<LOQ
dCTP	0.25±0.02	0.29±0.06	0.1±0.02	0.1±0	<LOQ	<LOQ	<LOQ	<LOQ	965±23.8	964.5±25.2
UTP	95.4±7.5	94.7±6.4	59±18.7	56.7±17.5	7.2±2.3	7.3±2.4	0.18±0.01	0.16±0.02	5324.4±167.4	5183.1±236.9
ITP	<LOQ	16.2±2.7	<LOQ	<LOQ	<LOQ	<LOQ	<LOQ	<LOQ	<LOQ	<LOQ
dGTP	0.3±0	0.3±0	<LOQ	0.23±0.03	<LOQ	<LOQ	<LOQ	<LOQ	277.8±15.9	270.2±18.8
8-oxo-dGTP	<LOQ	<LOQ	<LOQ	<LOQ	<LOQ	<LOQ	<LOQ	<LOQ	<LOQ	<LOQ
CTP	69.5±9	71.6±9.8	4.3±1.2	4.4±1.3	0.72±0.18	0.76±0.19	0.06±0	0.07±0	3353.2±87.7	3328.7±59.1
GTP	295.1±30.5	295.6±29	213.1±59.7	212.8±58.9	21±3.4	20.8±3.3	0.53±0.02	0.53±0.01	11926±696.7	11965.9±734.8
Suc-CoA (30 mg)	0.18±0.02	-	-	-	-	-	-	-	-	-
Mal-CoA (30 mg)	0.75±0.10	-	-	-	-	-	-	-	-	-
ppGpp	<LOQ	<LOQ	<LOQ	<LOQ	<LOQ	<LOQ	<LOQ	<LOQ	<LOQ	<LOQ
pppGpp	<LOQ	<LOQ	<LOQ	<LOQ	<LOQ	<LOQ	<LOQ	<LOQ	<LOQ	<LOQ

Table S10 Concentrations of nucleotides and other phosphorylated metabolites in E. coli treated with mupirocin for 0, 2,5, and 10 minutes.The metabolites were quantified on 3-cm and 5-cm iHILIC-(P) Classic, PEEK, 50×2.1, 5 µm. Concentration displayed as mean concentration ± confidence interval at confidence level 0.95

	E. coli 0 min [µM]		E. coli 2 min [µM]		E. coli 5 min [µM]]		E. coli 10 min [µM]	
**metabolite**	**3-cm**	**5-cm**	**3-cm**	**5-cm**	**3-cm**	**5-cm**	**3-cm**	**5-cm**
3-dp-CoA	<LOQ	<LOQ	<LOQ	<LOQ	<LOQ	<LOQ	<LOQ	<LOQ
dTMP	<LOQ	136.7±52.8	<LOQ	48.2±3.1	<LOQ	53.1±4.6	<LOQ	59.8±1.4
cyclocreatine	<LOQ	<LOQ	<LOQ	<LOQ	<LOQ	<LOQ	<LOQ	<LOQ
creatine	2.5±0.5	2.7±0.2	1.8±0	2.7±0.2	1.7±0.5	2.9±0.5	1.2±0.1	2.7±0.1
c-GMP	<LOQ	<LOQ	<LOQ	<LOQ	<LOQ	<LOQ	<LOQ	<LOQ
dUMP	<LOQ	114±8.4	<LOQ	70.7±3.5	<LOQ	70.7±4.5	<LOQ	39.6±1.9
c-di-AMP	<LOQ	<LOQ	<LOQ	<LOQ	<LOQ	<LOQ	<LOQ	<LOQ
AMP	1042.3±102	1010.9±100.1	969.5±92.8	922.7±66.4	940.6±58.9	936.5±61.6	872.4±97.6	857.9±60.1
acetyl-CoA	1822.7±49.5	1756.3±27.1	1978.1±30.5	1915.6±25.2	2131.5±33.8	2156.1±15	2263.8±120	2267.8±125
UMP	402.6±51.4	405.2±35.2	468.6±51.3	433±36.4	600.6±24.4	605.4±29	677.8±40.3	688.3±53.2
AICAR	715.2±54.5	988.2±89.5	19.7±4.5	62.9±10.6	1.1±0	11.1±0.5	0.7±0.1	8.2±0.6
IMP	307.4±17.4	304.6±15.2	52.7±2	50±1.8	19.7±0.8	17±1.5	13.3±1.5	10.5±1
dGMP	4.6±0.6	5.5±0.4	3.6±0.1	5.5±0.2	3.1±0.2	6.2±0.4	2.8±0.3	5.6±0.1
CoA	<LOQ	<LOQ	<LOQ	<LOQ	<LOQ	<LOQ	<LOQ	<LOQ
CMP	302.7±4.3	215.1±42.9	469.2±19.5	314.5±45.6	711.7±15.7	483.5±24.1	967.2±77.7	834.9±66.3
P-creatine	<LOQ	<LOQ	<LOQ	<LOQ	<LOQ	<LOQ	<LOQ	<LOQ
ADP	2278.9±142.3	1821.1±129.6	1761.3±59	1419.4±41.4	1772.2±76.3	1462.9±101.6	1698.1±258.8	1318.9±201.3
GMP	342.9±36.6	354.6±37.8	230.5±19.6	230.4±21.5	181.1±15.9	194.3±17	155.7±16.8	167.4±21.8
dCDP	55.7±4.4	50.8±3.5	40.5±2.3	38.4±1.9	46.7±0	43.8±0.7	42.9±7.9	41.6±7.1
dATP	911.5±39.7	909.3±46.5	889±18	875.1±15	629.9±19.5	629.2±16.7	455±28.5	455.8±26.4
UDP-Glc	8104.5±292.6	8471.9±169.1	12032.3±540.4	12615±589.9	16833.4±123.4	17923.2±148.5	19236.2±1604.1	20080.1±1720.3
UDP	1029.9±182.9	1308.1±178	976±137.6	1294.2±104.8	1255.4±97.3	1714.3±87.1	1350.1±136.8	1866.8±174.2
dTTP	566.3±23.4	560.3±15.7	755.5±63	746±45.8	1106.1±8.4	1103.6±4.6	1291.5±66.5	1268.1±85.2
IDP	<LOQ	<LOQ	<LOQ	<LOQ	<LOQ	<LOQ	<LOQ	<LOQ
CDP	240.7±21.8	238.2±19.6	239.7±12.2	237.4±13.2	322.7±16.4	322.5±13.4	382.7±83.6	386.5±85.7
ATP	11839.3±323.7	12010.3±410.5	14151.4±384.4	14162.2±294	13914.6±415.6	14378.2±363.4	13079.3±803.9	13063.6±910.9
c-di-GMP	<LOQ	<LOQ	<LOQ	<LOQ	<LOQ	<LOQ	<LOQ	<LOQ
dUTP	<LOQ	<LOQ	<LOQ	<LOQ	<LOQ	<LOQ	<LOQ	<LOQ
dITP	<LOQ	<LOQ	<LOQ	<LOQ	<LOQ	<LOQ	<LOQ	<LOQ
GDP	1904.9±160.1	1910.1±138.5	926.2±57.1	922.9±49	737.2±76.1	723.5±66	594.7±108.5	597.4±116.2
8-oxo-dATP	<LOQ	<LOQ	<LOQ	<LOQ	<LOQ	<LOQ	<LOQ	<LOQ
dCTP	965±23.8	964.5±25.2	1204.6±29	1221.8±32.3	1469.3±26.3	1474.6±19.6	1276.2±90.6	1288.9±94.1
UTP	5324.4±167.4	5183.1±236.9	7401.8±404	7415.5±322.7	9788.3±458.8	9517.1±150.7	10550.2±1012.5	10044.1±631.2
ITP	<LOQ	<LOQ	<LOQ	<LOQ	<LOQ	<LOQ	<LOQ	<LOQ
dGTP	277.8±15.9	270.2±18.8	199.1±7.8	194.9±7.9	158.7±5.1	161.9±4.9	147.6±12.5	149.8±10.6
8-oxo-dGTP	<LOQ	<LOQ	<LOQ	<LOQ	<LOQ	<LOQ	<LOQ	<LOQ
CTP	3353.2±87.7	3328.7±59.1	5978.6±241.1	6011.3±224.6	8552.3±32.2	8590.7±59.8	9109.2±574.4	9191.7±613.7
GTP	11926±696.7	11965.9±734.8	10494.4 ± 608	10306.5±541.2	8863.9 ± 442.5	8839.8±445	7328 ± 560.5	7504.6±524.9
Suc-CoA	-	-	-	-	-	-	-	-
Mal-CoA	<LOQ	<LOQ	<LOQ	<LOQ	<LOQ	<LOQ	<LOQ	<LOQ
ppGpp	<LOQ	<LOQ	425.6±33.3	441±30.8	258.5±24.4	402.1±30.4	259.7±20.8	301.5±30.8
pppGpp	<LOQ	<LOQ	241±20.5	384.6±23.1	228.2±21.8	184.6±8.2	179.5±20.5	79.5±20.5

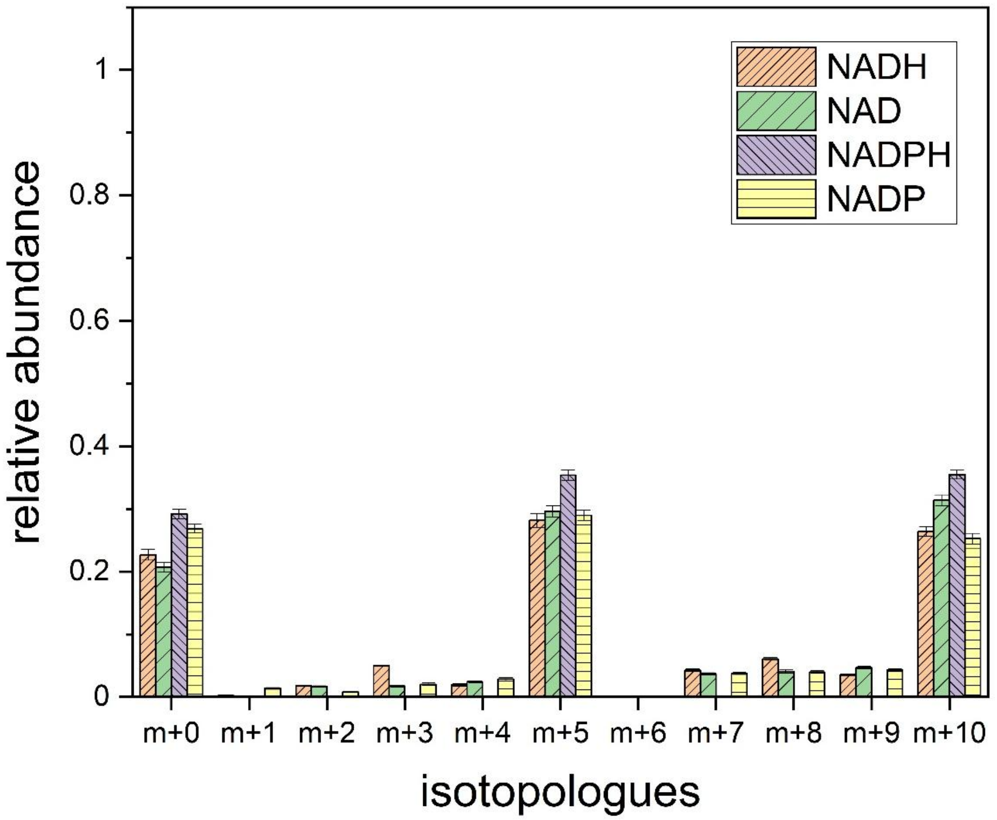

Figure S8 Relative abundance of isotopologues detected in NAD cofactors by LC-MS/MS (n = 3). Note that isotopologues between m+11 and m+21 were not detected. NADPH showed the least abundance in the samples and only m+5 and m+10 were detected.

Table S11 Distribution (%) of mono-, di-, and triphosphates in standard nucleotide samples dissolved in 50% aqueous methanol, water or 50 % aqueous acetonitrile stored in -20 °C, after multiple freeze-thaw cycles, measured by LC-MS.

Metabolite	ATP	ADP	AMP	CTP	CDP	CMP	GTP	GDP	GMP	UTP	UDP	UMP
**50% aqueous methanol**												
Freshly prepared solution	98	2	0	100	0	0	99	1	0	100	0	0
1. freeze-thaw cycle	98	2	0	99	1	0	99	1	0	100	0	0
2. freeze-thaw cycle	99	1	0	99	1	0	99	1	0	100	0	0
3. freeze-thaw cycle	99	1	0	99	1	0	99	1	0	100	0	0

10. freeze-thaw cycle	99	1	0	99	1	0	98	2	0	100	0	0
12. freeze-thaw cycle	99	1	0	99	1	0	98	2	0	100	0	0
14. freeze-thaw cycle	99	1	0	100	0	0	99	1	0	100	0	0

**Metabolite**	**ATP**	**ADP**	**AMP**	**CTP**	**CDP**	**CMP**	**GTP**	**GDP**	**GMP**	**UTP**	**UDP**	**UMP**
**Water solution**												
Freshly prepared solution	98	2	0	100	0	0	99	1	0	100	0	0
1. freez-thaw cycle	98	2	0	99	1	0	98	2	0	100	0	0
2. freez-thaw cycle	99	1	0	99	1	0	99	1	0	100	0	0
3. freez-thaw cycle	98	2	0	99	1	0	99	1	0	100	0	0
10. freez-thaw cycle	99	1	0	99	1	0	98	2	0	100	0	0
12. freez-thaw cycle	98	2	0	99	1	0	98	2	0	100	0	0
14. freez-thaw cycle	99	1	0	100	0	0	99	1	0	100	0	0
**50 % aqueous acetonitrile solution**												
Freshly prepared solution	98	2	0	100	0	0	99	1	0	99	1	0
1. freez-thaw cycle	98	2	0	99	1	0	98	2	0	100	0	0
2. freez-thaw cycle	98	2	0	99	1	0	97	3	0	100	0	0
3. freez-thaw cycle	98	2	0	99	1	0	98	2	0	100	0	0
10. freez-thaw cycle	99	1	0	99	1	0	93	7	0	100	0	0
12. freez-thaw cycle	99	1	0	99	1	0	98	2	0	100	0	0
14. freez-thaw cycle	99	1	0	100	0	0	98	2	0	100	0	0

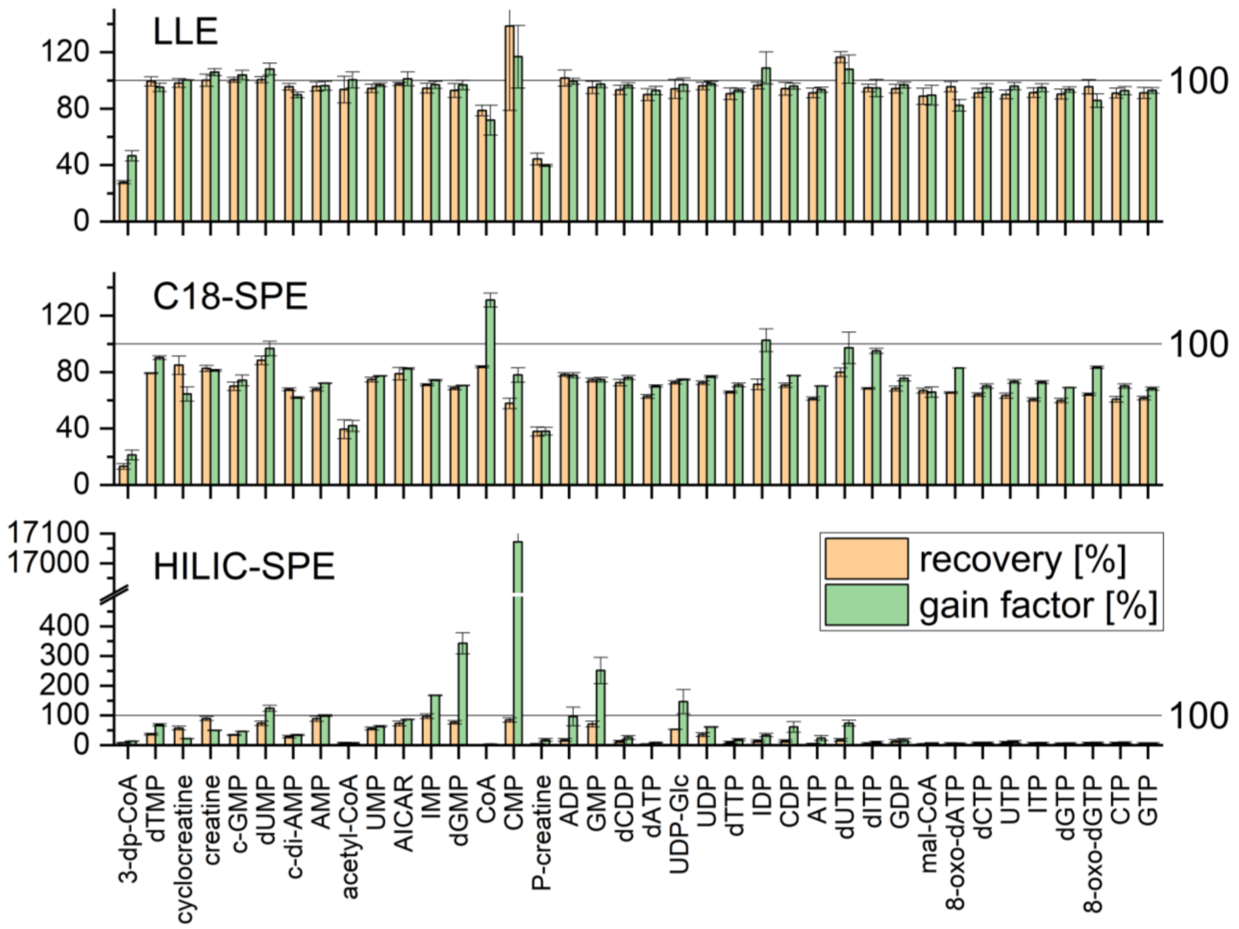

Figure S9 Recovery and gain factor calculated from spiked plasma samples analyzed by iHILIC - (P) Classic 50 × 2.1 mm, 5 µm

References

1. Varik, V., Oliveira, S. R. A., Hauryliuk, V. & Tenson, T. HPLC-based quantification of bacterial housekeeping nucleotides and alarmone messengers ppGpp and pppGpp. *Sci Rep* **7**, 11022 (2017).
2. Kanta, J. M. *et al.* Metabolic effects of medium-chain triacylglycerol consumption are preserved in obesity. *American Journal of Physiology-Endocrinology and Metabolism* **328**, E1–E20 (2025).
3. Miranda-Cervantes, A. *et al.* Pantothenate kinase 4 controls skeletal muscle substrate metabolism. *Nat Commun* **16**, 345 (2025).

4. Lindén, P., Keech, O., Stenlund, H., Gardeström, P. & Moritz, T. Reduced mitochondrial malate dehydrogenase activity has a strong effect on photorespiratory metabolism as revealed by^{13} C labelling. *EXBOTJ* **67**, 3123–3135 (2016).
5. Liebsch, D. *et al.* Metabolic control of arginine and ornithine levels paces the progression of leaf senescence. *Plant Physiology* **189**, 1943–1960 (2022).
6. ICH M10 on bioanalytical method validation - Scientific guideline | European Medicines Agency (EMA). https://www.ema.europa.eu/en/ich-m10-bioanalytical-method-validation-scientific-guideline (2019).
